# Critical contribution of the intracellular C-terminal region to TRESK channel activity is revealed by the epithelial Na^+^ current ratio method

**DOI:** 10.1016/j.jbc.2023.104737

**Published:** 2023-04-20

**Authors:** Dorina Debreczeni, Dóra Baukál, Enikő Pergel, Irén Veres, Gábor Czirják

**Affiliations:** Department of Physiology, Semmelweis University, Budapest, Hungary

**Keywords:** TRESK, K2P18, KCNK18, C-terminus, ENaC, K^+^ channel activity, Xenopus oocyte

## Abstract

TRESK (K_2P_18.1) possesses unique structural proportions within the K_2P_ background potassium channel family. The previously described TRESK regulatory mechanisms are based on the long intracellular loop between the second and the third transmembrane segments (TMS). However, the functional significance of the exceptionally short intracellular C-terminal region (iCtr) following the fourth TMS has not yet been examined. In the present study, we investigated TRESK constructs modified at the iCtr by two-electrode voltage clamp and the newly developed epithelial sodium current ratio (ENaR) method in *Xenopus* oocytes. The ENaR method allowed the evaluation of channel activity by exclusively using electrophysiology and provided data that are otherwise not readily available under whole-cell conditions. TRESK homodimer was connected with two ENaC (epithelial Na^+^ channel) heterotrimers, and the Na^+^ current was measured as an internal reference, proportional to the number of channels in the plasma membrane. Modifications of TRESK iCtr resulted in diverse functional effects, indicating a complex contribution of this region to K^+^ channel activity. Mutations of positive residues in proximal iCtr locked TRESK in low activity, calcineurin-insensitive state, although this phosphatase binds to distant motifs in the loop region. Accordingly, mutations in proximal iCtr may prevent the transmission of modulation to the gating machinery. Replacing distal iCtr with a sequence designed to interact with the inner surface of the plasma membrane increased the activity of the channel to unprecedented levels, as indicated by ENaR and single channel measurements. In conclusion, the distal iCtr is a major positive determinant of TRESK function.

Twik-Related Spinal cord K^+^ channel, K_2P_18.1, KCNK18 (TRESK) is a divergent member of the two-pore-domain (K_2P_) background potassium channel family (for reviews, see (1, 2, 3, 4, 5)). It is the single representative of the TRESK subgroup, unequivocally distinguishable from the other K_2P_ channels in the species from fish to human (6). In mammals, TRESK is highly expressed in particular subpopulations of primary sensory neurons (7, 8) and also at a lower level in different structures of the central nervous system (9) and in other tissues (10, 11, 12). The function of this channel is currently being elucidated. TRESK has been reported to contribute to the control of pain sensation and prevention of migraine (13, 14, 15, 16, 17, 18, 19), intellectual function (20, 21, 22), depolarization-induced shunting inhibition in epilepsy (23), regulation of circadian rhythm in the suprachiasmatic nucleus (24), and the development of regulatory T cells in the immune system (25).

TRESK shares low (<30%) amino acid identity with the other members of the K_2P_ family (26). The sequence similarity is confined to the transmembrane segments (TMSs), and the intracellular domains are different from the other channels. Accordingly, TRESK has special regulatory features (1), for example, it is activated by calcineurin-dependent dephosphorylation (10). In addition to the divergent sequences, the proportions of intracellular regions also deviate from the other K_2P_ channels (Fig. 1). The unusually long intracellular loop between the second and third TMSs, 121 amino acids in human TRESK, contains the currently known regulatory motifs. This includes the calcineurin binding sites (27, 28), and the serine residues S262 and S264 dephosphorylated by this calcium/calmodulin-dependent protein phosphatase (10, 29). In sharp contrast, the intracellular C-terminal region (iCtr) following the fourth TMS, which is long in the other K_2P_ channels, is exceptionally short in TRESK, with only 29 amino acids. The major objective of the present study was to investigate whether this short iCtr contributes to TRESK channel activity.

**Figure 1:**
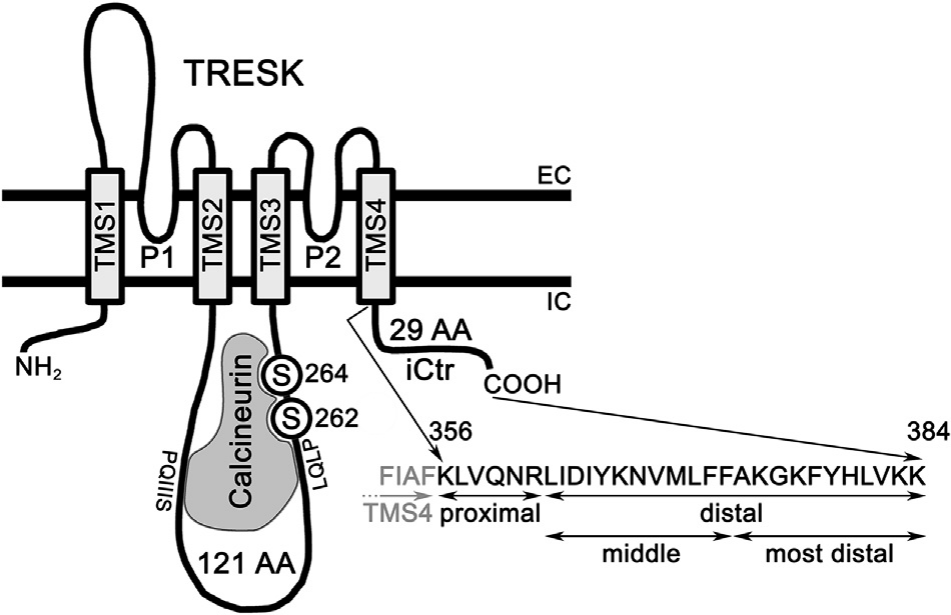
**Schematic membrane topology of human TRESK.** TRESK functions as a homodimer of subunits. In this figure, one subunit is illustrated, which follows the general 4TMS/2P molecular architecture of K_2P_ channels; it possesses four TMS1-4 and two pore domains (P1-2). However, the proportions of intracellular regions are unique in TRESK. The long intracellular loop (121 amino acids) contains the known regulatory elements. For simplicity, only calcineurin and its binding sites (the *PQIIIS* and *LQLP* sequences) and the dephosphorylated regulatory serine residues (*S262*,*S264*) are shown. The iCtr is exceptionally short, with only 29 amino acids. In accordance with its fundamental and diverse functions, it is practical to divide the iCtr into proximal and distal parts, the latter including the middle and most distal regions. Considering the final conclusions of the present study, the proximal part can also be regarded as a “gating” domain. The middle part contains two hydrophobic stretches (LIDIY and VMLFF), whereas the most distal part could be called an “interaction” domain. (The two thick horizontal lines represent the plasma membrane; EC is extracellular and IC is intracellular. The scheme is not drawn to scale). iCtr, intracellular C-terminal region; TASK, TWIK-related Acid-Sensitive K+ channel; TEVC, two-electrode voltage clamp; TMS, transmembrane segment; TRESK, TWIK-Related Spinal cord K+ channel

Conventional whole-cell electrophysiology results in an irrevocable loss of information. Currents and their regulation by signaling, or pharmacological modulation, are precisely measured; however, no direct data about the channel activity is obtained because the number of channels in the plasma membrane is not determined. This problem is especially conspicuous when mutants of deficient regulation are studied. Then a constant current is recorded, which may correspond either to a high number of low-activity or to a low number of high-activity channels. The expression level is usually unknown, as it can also be strongly influenced by the mutation itself. Specific approaches have been adapted to overcome this problem, for example, the extracellular tagging of the ion channel with an epitope, and immunodetection of the protein after the electrophysiology, followed by luminometry (30, 31, 32, 33). However, these methods are not generally applied, since the expression of the channel protein is typically low, it is at the limit of detection, and the estimation of the number of channels requires lengthy post-processing of the cell after the current measurement.

We have developed the ENaR method to gain insight into the K^+^ channel activity in *Xenopus* oocytes, by exclusively using current measurements. In simple terms, the different TRESK mutants had been tagged with two ENaC channels, and the K^+^ and Na^+^ currents were measured separately in the same cell. The K^+^ current/Na^+^ current ratio is determined as the ENaR value, a parameter proportional to the activity level of the mutant TRESK channel. The observation of ENaR values raised ideas, which would not have emerged from the conventional whole-cell electrophysiology.

## Results

### ENaR data are consistent with the known relationships of channel activity of the phospho- and dephospho-mimicking TRESK mutants

**Figure 2:**
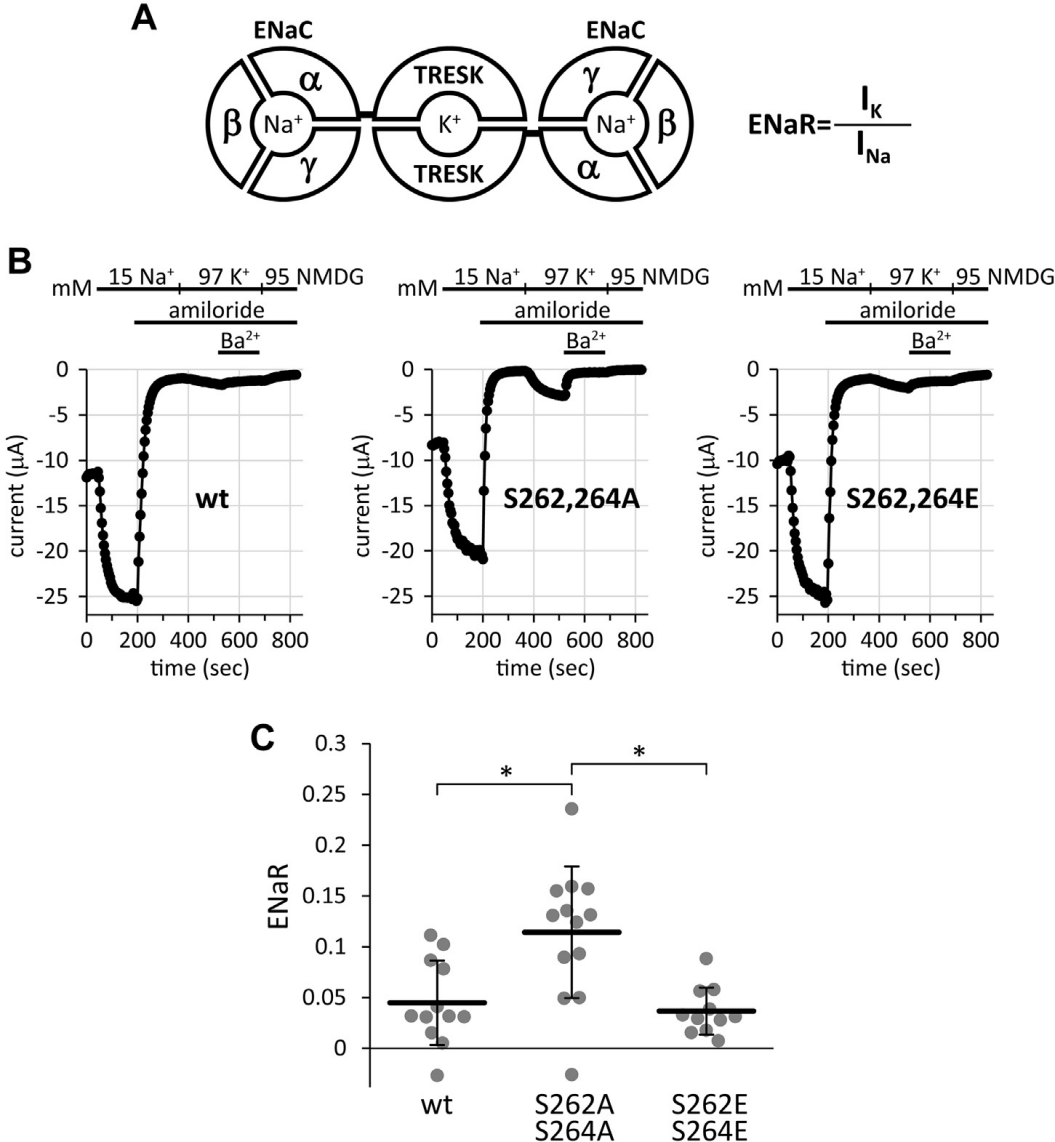
**Analysis of phospho- and dephospho-mimicking TRESK mutants by the ENaR method.***A*, cartoon representation of the protein complex expressed in ENaR experiments. The ENaCα-TRESK fusion protein co-assembles with the ENaC β and γ subunits, and constitutes one TRESK and two ENaC pores. (All ENaC subunits were truncated in order to eliminate regulatory sequences). The ENaR value in a cell is defined as the ratio of K^+^ to Na^+^ current through this protein complex (in fraction form). *B*, recording protocol for the consecutive measurement of ENaC Na^+^ and TRESK K^+^ currents in three representative *Xenopus* oocytes in an ENaR experiment. In the *left panel*, the ENaCα-TRESK fusion construct, containing the wild-type TRESK sequence (*wt*), is coexpressed with ENaC β and γ. In the *middle panel*, ENaCα-TRESK carries the dephospho-mimicking S262A and S264A (*S262*,*264A*), whereas in the *right panel*, the phospho-mimicking S262E and S264E mutations (*S262*, *264E*). ENaC and TRESK currents were measured as the Na^+^ current inhibited by amiloride (20 μM), and the K^+^ current inhibited by Ba^2+^ (5 mM), respectively, as indicated by the *horizontal black bars*. Note that the K^+^ current is relatively large in proportion to the Na^+^ current in the case of the S262, 264A mutant. *C*, statistical analysis of the ENaR values in three groups of oocytes expressing the constructs introduced in panel *B* (and indicated below the *graph*). Each *grey circle* represents the ENaR value of a single cell. The *horizontal black lines with error bars* indicate the average ENaR ± SD. ∗*p* < 0.005, one-way ANOVA, and Tukey HSD test (n = 12, 13, and 11, respectively). ENaC, epithelial Na+ channel; ENaR, epithelial sodium current ratio (method or value); TMS, transmembrane segment; TRESK, TWIK-Related Spinal cord K+ channel.

Human TRESK protein has been connected to the C-terminus of a truncated version of mouse epithelial Na^+^ channel (ENaC) α in order to form a single polypeptide chain from the two unrelated subunits. This ENaCα-TRESK fusion construct was coexpressed with excess amounts of the truncated ENaC β and γ in *Xenopus* oocytes. A possible protein complex resulting from the coexpression is illustrated in Figure 2*A*. This stoichiometric complex contains two Na^+^-selective ENaC pores in addition to the TRESK K^+^ channel.

The Na^+^ and K^+^ current components have been measured in each oocyte separately, as the amiloride-sensitive Na^+^ current in 15 mM EC [Na^+^], and the Ba^2+^-sensitive K^+^ current in 97 mM EC [K^+^] (Fig. 2*B*). By dividing the K^+^ current with the Na^+^ current, an ENaR value has been assigned to each cell, reflecting TRESK activity (ENaR = I_K_/I_Na_). Wild-type TRESK is phosphorylated and inhibited under resting conditions (10, 27, 28, 29, 34); therefore, the K^+^ currents and the ENaR values are small in the wild-type group (Fig. 2*B*, *left panel*). If the G_q_-protein-coupled M_1_ muscarinic acetylcholine receptor was also coexpressed and stimulated with carbachol, then TRESK, in complex with ENaC, was activated in response to the calcium signal (Fig. S1*A*, see the “S” figures in the Supporting information).

In human TRESK, serine residues 262 and 264 are the major functional determinants of calcineurin-dependent regulation (10, 34). The S262A and S264A mutants correspond to the dephosphorylated active state of the channel, whereas the replacement of these serines with negatively charged glutamates, S262E and S264 E, imitates the inhibitory phosphorylation. When the highly active, dephospho-mimicking S262,264A double mutant was connected to the truncated ENaC α subunit, and coexpressed with the truncated ENaC β and γ, the K^+^ current was indeed relatively large, and represented a higher fraction, compared to the sodium current (Fig. 2*B*, *middle panel*). In contrast, the phospho-mimicking S262,264E mutant caused relatively small K^+^ currents in the ENaR experiments (Fig. 2*B*, *right panel*), similar to the wild-type channel under basal conditions. Accordingly, the ENaR values of the S262,264A group were significantly higher than those of the wild type or S262,264E channels (Fig. 2*C*). In another experiment, the average ENaR of the S262,264A mutant was similar to that of the wild-type channel after the activation *via* the M_1_ receptor, and much higher than the basal ENaR of the wild-type TRESK, or that of the other phospho-mimicking S262,264D mutant (Fig. S1, *B* and *C*). Altogether, the ENaR data correspond well to the expected relationships of activity in the case of the extensively investigated calcineurin-dependent regulation of TRESK. The ENaR method has the potential for the analysis and comparison of the channel activities of different TRESK mutants.

### Basic properties and limitations of the ENaR system

The coexpression of the truncated ENaC β and γ subunits did not result in a Na^+^ current on the μA scale, in the absence of the α subunit (Fig. S2). Therefore, the ENaCα-TRESK fusion construct is an obligate element of the functional protein complex in the ENaR experiments. When ENaCα-TRESK was coexpressed with the truncated ENaC β subunit, in the absence of ENaC γ, then the Na^+^ current similarly disappeared, and the TRESK current was also lost (Fig. S3). This indicates that the expression of ENaCα-TRESK does not result in an independent TRESK current component in the absence of the ENaC heterotrimer.

**Figure 3:**
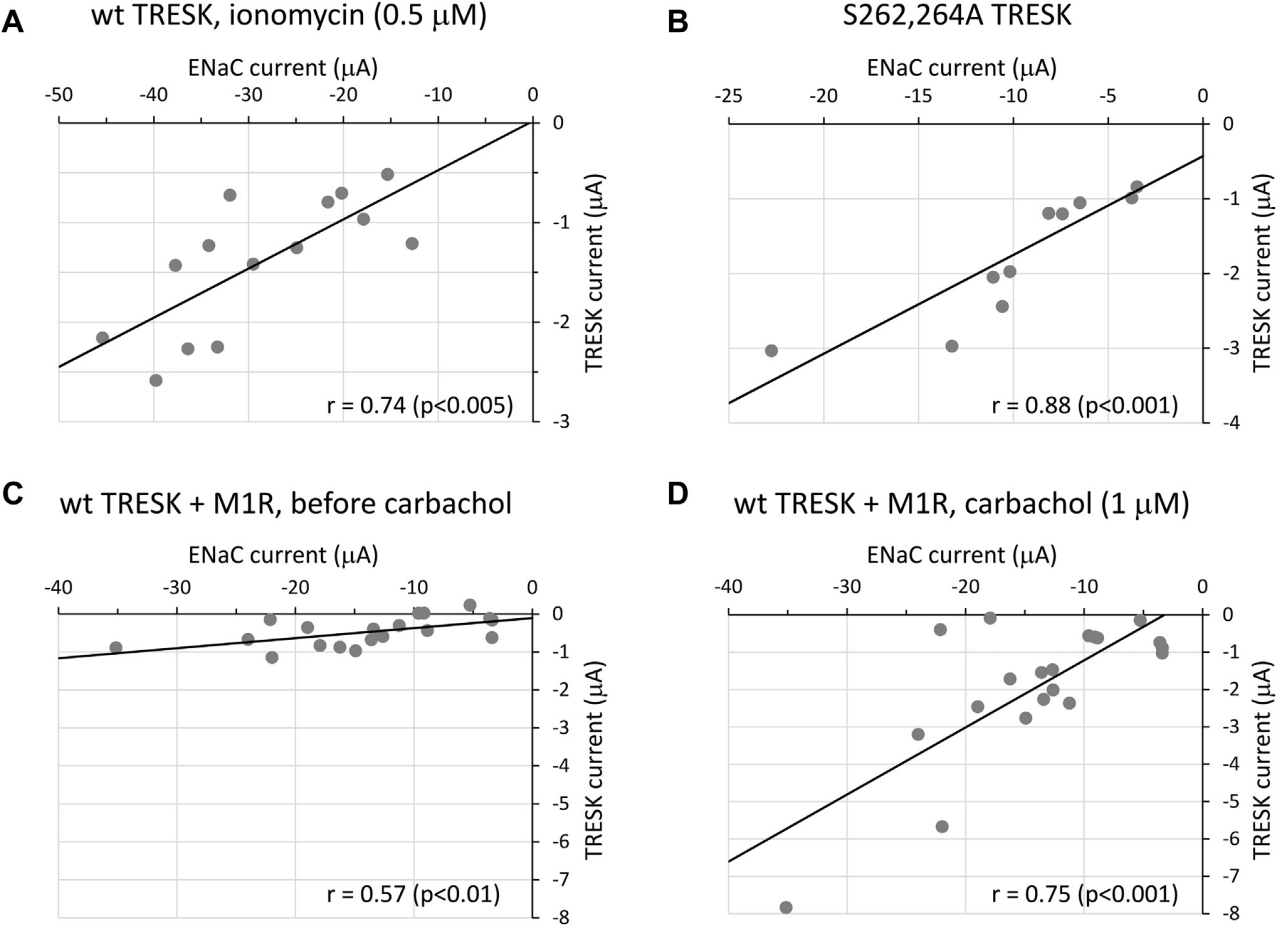
**Correlation analysis of TRESK *versus* ENaC currents in ENaR experiments under different experimental conditions.***A*, *Xenopus* oocytes coexpressing ENaCα-TRESK with ENaC β and γ were stimulated with ionomycin in order to activate wild-type TRESK, which had been built in the fusion construct. The amiloride-sensitive Na^+^ current was measured before, while the Ba^2+^-sensitive K^+^ current after the stimulation. K^+^ currents were plotted against Na^+^ currents, and each *gray circle* indicates a cell in the *graph*. Pearson’s correlation coefficient (*r*) is shown in the *lower right corner*. *B*, similar correlation analysis as in panel *A*, applying the constitutively active, S262A + S264A double mutant ENaCα-TRESK. *C*, In this experiment, M_1_ muscarinic receptor was also coexpressed with ENaCα-TRESK, ENaC β and γ. Before receptor stimulation, the correlation was weak between the small TRESK and robust ENaC currents. *D*, M_1_ receptor in the same cells as in panel *C* was stimulated with carbachol. TRESK was activated and the correlation improved. (Two cells did not respond to the stimulation). ENaC, epithelial Na+ channel; ENaR, epithelial sodium current ratio (method or value); TMS, transmembrane segment; TRESK, TWIK-Related Spinal cord K+ channel

The K^+^ currents show reasonable correlations with the Na^+^ currents in the ENaR experiments, however, the Pearson’s coefficients are not precisely equal to one (Fig. 3). The functional state of the K^+^ and Na^+^ channels in the protein complex may vary, for example, the degree of regulatory TRESK phosphorylation may fluctuate from cell to cell. Accordingly, ENaR has to be determined as an average from several cells. For the same reason, the ENaR of the oocytes expressing the same construct may be variable in the different cell preparations. However, in this case, the relationships between the ENaR averages of the different TRESK constructs are reproducible.

A more general application of the ENaR system is possible than the investigation of TRESK mutants. The activity of other ion channels can also be examined, or even compared under identical conditions. For example, the members of the TWIK-related Acid-Sensitive K+ channel (TASK) subfamily of K_2P_ channels produced remarkably high basal ENaR values. Mouse TASK-3 significantly outperformed human TASK-1 in this respect (Fig. S4).

### Progressive deletions of the iCtr diminish TRESK channel activity

**Figure 4:**
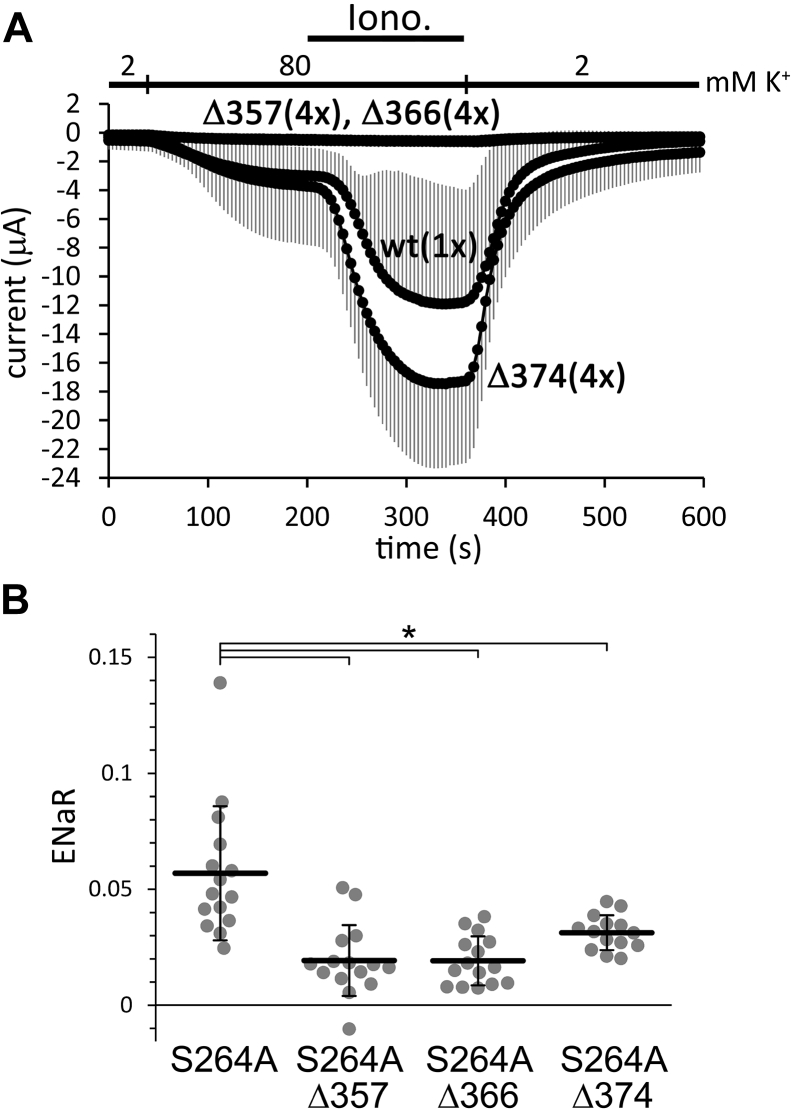
**Deletions of the iCtr decrease TRESK expression and/or channel activity.***A*, conventional TEVC measurement of four groups of oocytes expressing wild type (*wt*), *Δ357*, *Δ366*, or *Δ374* TRESK, (n = 10, 6, 6, and 10), respectively. Average currents ± SD are plotted. Four-times (*4×*) more cRNA was microinjected in the groups of truncated TRESK channels than in the wild-type control (*1×*). The cells were stimulated with ionomycin (*Iono.*, 0.5 μM), as indicated by the *black bar*. The Δ357 and Δ366 channels are not expressed and these curves overlap. *B*, ENaR analysis of the deletions in the constitutively active S264A ENaCα-TRESK (see the groups *below the panel*). The method of measurement and analysis was the same as in Figure 2. ∗*p* < 2 × 10^−4^, one-way ANOVA, Tukey HSD test (n = 15, 15, 15, and 14, respectively). iCtr, intracellular C-terminal region; ENaC, epithelial Na+ channel; ENaR, epithelial sodium current ratio; TRESK, Twik-Related Spinal cord K+ channel, K2P18.1, KCNK18.

The iCtr was truncated in three consecutive steps; 11, 19, or 28 amino acids were deleted in the Δ374, Δ366, and Δ357 constructs, respectively (see Table 1). In conventional two-electrode voltage clamp (TEVC) measurements, the expression of the Δ357 and Δ366 mutants did not result in K^+^ currents, even if four times more cRNA was microinjected than that of the wild-type control, and the cells were stimulated with ionomycin (Fig. 4*A*). The fourfold cRNA quantity of Δ374 resulted in similar basal K^+^ currents as the wild-type TRESK, and the current of the Δ374 construct was activated normally with ionomycin (Fig. 4*A*).

**Table 1:** Amino acid sequences of the intracellular C-terminal region (iCtr) in the different TRESK constructs

Construct	Amino acid sequence
wt	...FIAFKLVQNRLIDIYKNVMLFFAKGKFYHLVKK
Δ374	...FIAFKLVQNRLIDIYKNVMLFF
Δ366	...FIAFKLVQNRLIDI
Δ357	...FIAFK
K356A	...FIAF**A**LVQNRLIDIYKNVMLFFAKGKFYHLVKK
R361A	...FIAFKLVQN**A**LIDIYKNVMLFFAKGKFYHLVKK
K367A	...FIAFKLVQNRLIDIY**A**NVMLFFAKGKFYHLVKK
K356A, R361A	...FIAF**A**LVQN**A**LIDIYKNVMLFFAKGKFYHLVKK
K356E	...FIAF**E**LVQNRLIDIYKNVMLFFAKGKFYHLVKK
R361E	...FIAFKLVQN**E**LIDIYKNVMLFFAKGKFYHLVKK
K356E, R361E	...FIAF**E**LVQN**E**LIDIYKNVMLFFAKGKFYHLVKK
RAADAA	...FIAFKLVQNR**AA**D**AA**KNVMLFFAKGKFYHLVKK
VMLFF-5A	...FIAFKLVQNRLIDIYKN**AAAAA**AKGKFYHLVKK
F372L	...FIAFKLVQNRLIDIYKNVML**L**FAKGKFYHLVKK
4K4W2K	...FIAFKLVQNRLIDIYKNVMLFFA**KKKKWWWWKK**
12K	...FIAFKLVQNRLIDIYKNVMLFFA**KKKKKKKKKKKK**
12N	...FIAFKLVQNRLIDIYKNVMLFFA**NNNNNNNNNNNN**
12D	...FIAFKLVQNRLIDIYKNVMLFFA**DDDDDDDDDDDD**
375-6Kcaax	...FIAFKLVQNRLIDIYKNVMLFFA**KKKKKKSKTKCVIM**
367-6Kcaax	...FIAFKLVQNRLIDIY**KKKKKKSKTKCVIM**
356-6Kcaax	...FIAF**KKKKKKSKTKCVIM**
RW361	...FIAFKLVQN**RLLRLLRRLLLWWGKKKKKK**

The effect of deletions was examined in the context of the S264A activating mutation in ENaR experiments (Fig. 4*B*). All three truncated constructs, S264A + Δ357, S264A + Δ366, and S264A + Δ374 induced sufficient Na^+^ current for the analysis (17.0 ± 6.1, 16.6 ± 6.7, and 18.3 ± 5.6 μA; n = 15, 15, and 14, respectively), indicating that ENaC facilitated the functional expression of the Δ357 and Δ366 mutants. The average ENaRs of all three truncated constructs were significantly lower than that of the positive control S264A mutant, indicating that the intact iCtr is indispensable for the generation of high TRESK channel activity.

In the presence of S264A activating mutation, the deletion of the most distal iCtr, the "interaction" domain (see Fig. 1), resulted in functional channels showing lower activity than the wild type. However, the deletion of the second hydrophobic motif (VMLFF) in addition to the "interaction" domain, as well as the deletion of the "gating", hydrophobic, and "interaction" domains together, reduced the activity to very low levels. (In some experiments we used the S264A mutant, whereas in others the S262,264A double mutant TRESK as the constitutively active control. For the comparison of these mutants, see Fig. S5).

### The mutations of K356 and R361 trap TRESK in a low activity, unregulated state

**Figure 5:**
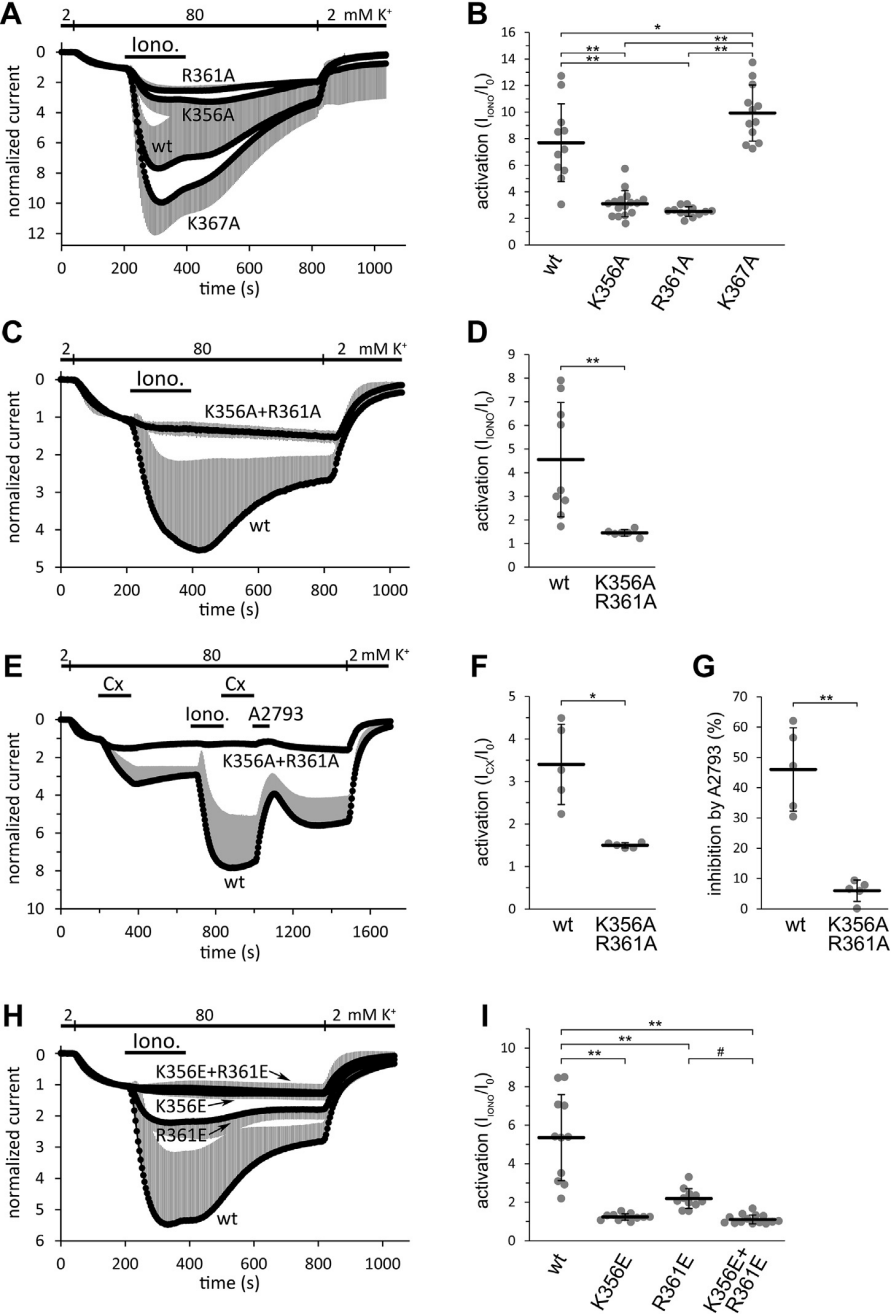
**Conventional TEVC measurements of TRESK mutated at the K356, R361, or K367 residues.***A*, normalized K^+^ currents of four groups of oocytes expressing wild type (*wt*), *K356A*, *R361A* or *K367A* mutant TRESK, respectively. TRESK currents were measured at the ends of 300-ms voltage steps to −100 mV applied every 4 s. Extracellular [K^+^] was increased from 2 to 80 mM (as shown *above the curves*), and the oocyte was then challenged with ionomycin (*Iono.*, 0.5 μM) as indicated by the *horizontal black bar*. The current measured in 2 mM EC [K^+^] was normalized to zero, and the current in 80 mM EC [K^+^], before the application of ionomycin, to one. The *grey error bars* represent S.D. For average current data corresponding to *panels A*, *C*, *E*, and *H*, see Fig. S6. *B*, Statistical analysis of TRESK activation by ionomycin from the same groups as shown in panel *A* (and indicated *below the graph*). The maximum current during the stimulation with ionomycin was divided by the resting value before the application of the ionophore (I_IONO_/I_0_), and the degree of activation was plotted for each cell as a *grey circle*. ∗*p* < 0.03, ∗∗*p* < 0.005, one-way ANOVA, Tukey HSD test (n = 11, 16, 12, and 12, respectively). *C*, the activation of the K356A + R361A double mutant TRESK by ionomycin was measured with the same protocol as in panel *A*, in a different cell preparation. *D*, statistical analysis of the activation by ionomycin in the wild type (*wt*) and *K356A + R361A* groups shown in panel *C*. ∗∗*p* < 0.005, Student’s *t* test (n = 9, and 6, respectively). *E*, normalized currents of oocytes expressing wild type (*wt*), or *K356A + R361A* TRESK, in a different cell preparation (n = 5 in both groups). Cloxyquin (*CX*, 100 μM) was applied before and after ionomycin (*Iono.*, 0.5 μM) as indicated by the *horizontal black bars*. TRESK inhibitor *A2793* (50 μM) was added after the second application of cloxyquin. *F*, statistical analysis of the activation by cloxyquin, using the data shown in panel *E*. The maximum K^+^ current in the presence of cloxyquin was normalized to the basal level measured before the administration of the compound (I_CX_/I_0_), and the degree of activation was plotted for each cell as a *grey circle*. ∗*p* < 0.02, Student’s *t* test. *G*, statistical analysis of the inhibition by A2793, using the data shown in panel *E*. The inhibition was calculated as a percentage, the inhibited current was compared to the amplitude measured before the addition of A2793 (*i.e.*, after the second application of cloxyquin). ∗∗*p* < 0.005, Student’s *t* test. *H*, Normalized K^+^ currents of four groups of oocytes expressing wild type (*wt*), *K356E*, *R361E* or *K356E + R361E* double mutant TRESK, respectively. The measurement was similarly performed as in panel *A*, but in a different cell preparation. The K356E and K356E + R361E curves overlap. *I*, Statistical analysis of TRESK activation by ionomycin from the same groups as shown in panel H (and indicated *below the graph*). The method of analysis was the same as in panel B. ∗∗*p* < 2 × 10^−4^, #*p* = 0.076, one-way ANOVA, Tukey HSD test (n = 11, 11, 11, and 15, respectively). TEVC, two-electrode voltage clamp; TRESK, Twik-Related Spinal cord K+ channel.

Mutational analysis indicated the functional relevance of the positively charged K356 and R361 amino acids in the proximal iCtr, which is located close to intracellular end of the fourth TMS (for sequence data, see Table 1). The K356A and R361A mutations diminished the calcium-dependent activation of TRESK, evoked by the calcium-ionophore ionomycin, as determined by conventional TEVC measurements (Fig. 5, *A* and *B*). The K367A mutation in the middle part of the iCtr did not result in a similar reduction of activation. The K356A + R361A double mutation decreased the effect of ionomycin to negligible levels (Fig. 5, *C* and *D*). Thus, the mutations of the proximal iCtr prevented TRESK regulation by calcineurin, although this phosphatase binds at a distant location and dephosphorylates serine residues in the intracellular loop of the channel (10, 34).

Cloxyquin and A2793 are state-dependent pharmacological modulators, the activator and inhibitor of TRESK, respectively (35, 36). Accordingly, the basal current of the wild-type channel was increased about 3-fold by cloxyquin, whereas this activator did not change the K^+^ current after the application of ionomycin (Fig. 5*E*, *wt curve*). The cloxyquin derivative A2793 inhibited the current of wild-type TRESK after ionomycin. In sharp contrast, the current of the K356A + R361A mutant TRESK was not affected by any of these interventions; ionomycin, cloxyquin, and A2793 failed to influence the current (Fig. 5, *E*–*G*). The effect of cloxyquin is independent of ionomycin, in the sense that this direct channel activator does not act *via* calcineurin (37). However, the K356A + R361A mutation also prevented the effect of cloxyquin in addition to ionomycin. This indicates that the K356A + R361A mutant cannot be activated by two independent mechanisms.

The charge-reversal mutations of the same residues, K356E, R361E, and K356E + R361E similarly abrogated the response to ionomycin (Fig. 5, *H* and *I*). The K356E and K356E + R361E mutations completely prevented the calcium-dependent activation.

**Figure 6:**
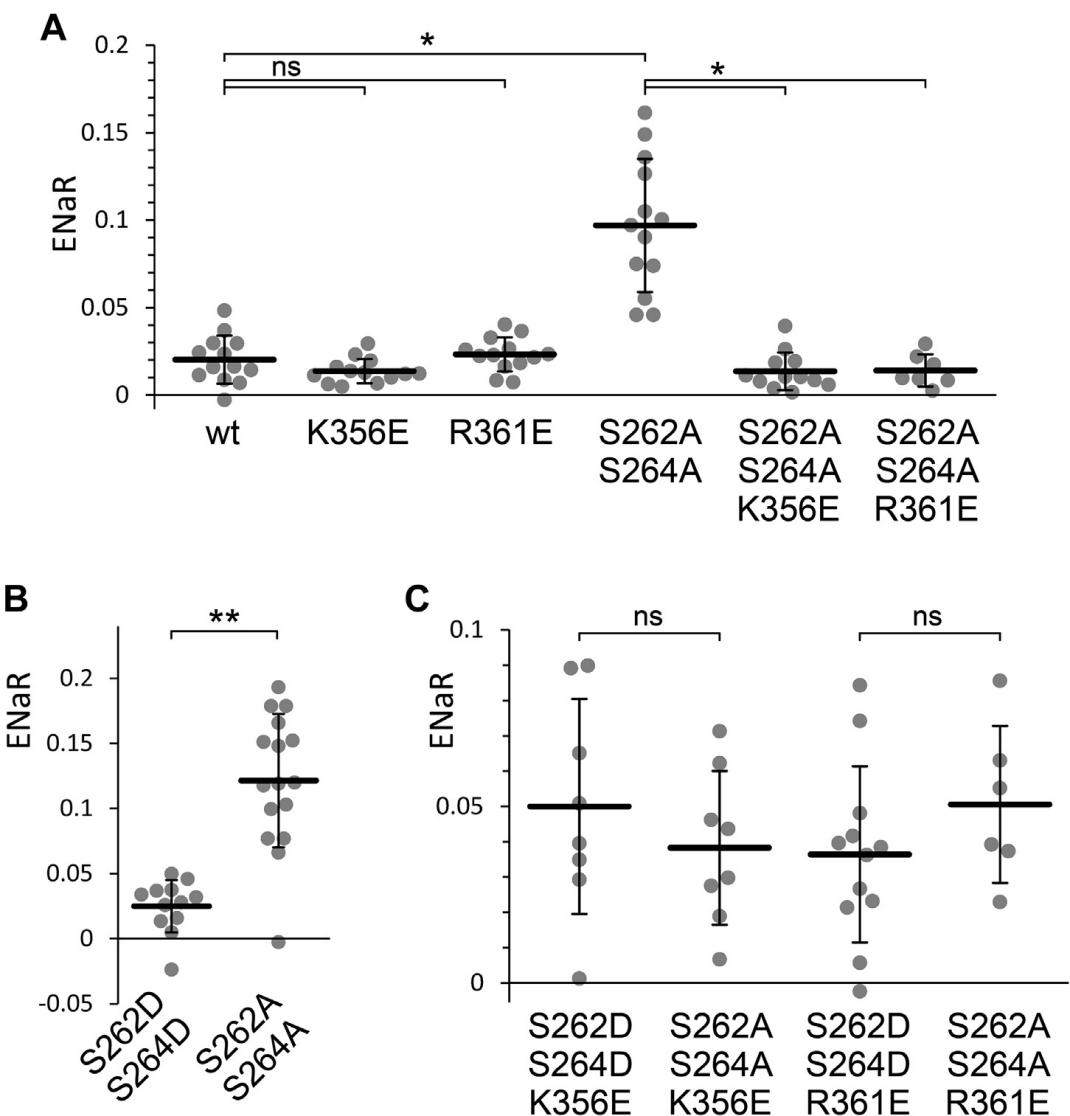
**ENaR analysis of the K356E and R361E mutations in the wild type, constitutively active S262,264A, and inhibited S262,264D ENaCα-TRESK channels.***A*, ENaR values are plotted from six groups of oocytes expressing ENaCα-TRESK with or without the S262A + S264A double mutation, and containing wild type, K356E, or R361E mutant versions of the iCtr, as indicated *below the graph*. The method of measurement (coexpression of truncated ENaC β and γ, amiloride- and Ba^2+^-sensitive currents, etc.) and analysis was the same as introduced in Figure 2. ∗*p* < 2 × 10^−4^, ns: not significant, one-way ANOVA, Tukey HSD test (n = 13, 13, 13, 13, 12 and 7, respectively). *B*, comparison of the ENaR values of the phospho-mimicking S262D + S264D and the dephospho-mimicking S262A + S264A double mutants of ENaCα-TRESK. Data are repeated in Fig. S1*C*. ∗∗*p* < 10^−6^, Student’s *t* test (n = 12, and 16, respectively). *C*, the missing effect of the S262D + S264D *versus* S262A + S264A mutations on the ENaR values in the K356E and R361 mutant ENaCα-TRESK channels. Groups are indicated *below the graph*. Note the different vertical scales in panels *B* and *C* ns: not significant, one-way ANOVA, Tukey HSD test (n = 8, 8, 12, and 6, respectively). (Panels *A*, *B*, and *C* correspond to three different cell preparations, respectively). ENaC, epithelial Na+ channel; ENaR, epithelial sodium current ratio; iCtr, intracellular C-terminal region; TRESK, Twik-Related Spinal cord K+ channel, K2P18.1, KCNK18.

It is challenging to differentiate, based on conventional whole-cell measurements, whether the above mutants are low-activity channels with a defective activation mechanism, or constitutively active constructs, which cannot be further activated. Nevertheless, the low efficiency of A2793 on the K356A + R361A mutant is in favor of the first option (Fig. 5*G*). In order to examine this question more adequately, the activity levels of K356E and R361E mutants were estimated in ENaR measurements (Fig. 6). The ENaRs of the K356E and R361E mutants were not significantly different from the wild-type channel, which is phosphorylated and has low activity under resting conditions (Fig. 6*A*, *left three groups*). When the K356E or R361E mutations were introduced in the dephospho-mimicking, constitutively active S262,264A background, then this modification of the iCtr decreased the channel activity, approximately to the resting level (Fig. 6*A*, *right three groups*). This indicates that the constitutive activity induced by the S262,264A mutations cannot prevail in the presence of K356E or R361E. (It is important to emphasize that the K356E mutation does not eliminate TRESK function; the K356E mutant TRESK also induces substantial K^+^ currents when it is overexpressed, see Fig. S6*D*. Instead, the low ENaR of the S262,264A + K356E construct means that the K356E mutation prevents the activation by the S262,264A mutation).

In another experiment, the S262,264D mutant was used as the low-activity control instead of the wild-type channel. The ENaR of the dephospho-mimicking S262,264A mutant is much higher than that of the phospho-mimicking S262,264D (Fig. 6*B*). However, this substantial difference between S262,264D and S262,264A disappears in the presence of the K356E or R361E mutations, and all four constructs show similar activity levels (Fig. 6*C*). Consequently, the K356E and R361E mutations disrupt the modulation of channel activity by the mutations of S262 and S264.

The missing effects of ionomycin, cloxyquin, A2793, and the S262,264A *versus* S262,264D mutations on the channel activity suggest that the mutations of K356 and R361 generally interfere with the transmission of modulation to the gating machinery of TRESK.

### The hydrophobic interactions of the iCtr are required for the high TRESK channel activity

**Figure 7:**
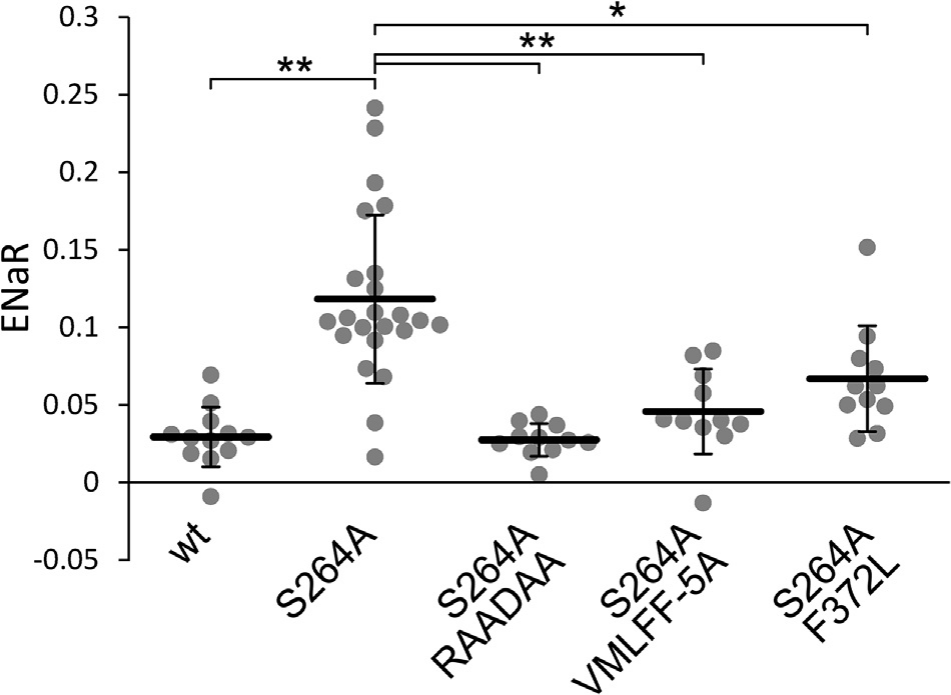
**Elimination of the hydrophobic modules of the distal iCtr and the F372L mutation reduces the ENaR of the constitutively active S264A ENaCα-TRESK.** ENaR values are plotted from five groups of oocytes expressing wild type (*wt*, negative control), *S264A* (positive control), or three other modified versions of S264A ENaCα-TRESK, containing the replacement of the RLIDIY sequence with *RAADAA*, the substitution of VMLFF sequence with five alanines (*VMLFF-5A*), or the *F372L* mutation, respectively, as indicated *below the graph*. The method of measurement was the same as in Figure 2. ∗*p* < 0.005, ∗∗*p* < 2 × 10^−4^, one-way ANOVA, Tukey HSD test (n = 12, 23, 11, 11, and 11, respectively). ENaC, epithelial Na+ channel; ENaR, epithelial sodium current ratio; iCtr, intracellular C-terminal region; TRESK, Twik-Related Spinal cord K+ channel, K2P18.1, KCNK18.

The contribution of two hydrophobic groups of amino acids to the maintenance of high channel activity was tested in the S264A context, by the RLIDIY to RAADAA and VMLFF to AAAAA mutations of the iCtr (see the RAADAA and VMLFF-5A constructs in Table 1). The elimination of the hydrophobic side chains in these regions significantly decreased the ENaR values of the constitutively active S264A channel (Fig. 7). The data suggest that the hydrophobic interactions are important in the function of the iCtr to maintain the high activity of the K^+^ channel.

The F372L mutation of human TRESK was reported in a patient with migraine (38), and it can also be found in the single-nucleotide polymorphism database, as the rare variant rs143365824. This mutation changes the first phenylalanine in the VMLFF motif to leucine, which partitions substantially less to the bilayer interface from water than the aromatic residue (39). We were curious whether the functional consequence of the F372L mutation on TRESK activity can be detected under the conditions of an ENaR experiment. The ENaR of the S264A + F372L mutant TRESK was significantly lower than that of the S264A control, suggesting that the F372L mutation decreased the K^+^ channel activity (Fig. 7). The relatively large effect of the F372L single mutation (compare the S264A + F372L to the S264A and S264A + VMLFF-5A groups in Fig. 7) raised the possibility that the phenylalanine has a prominent role in the overall function of the VMLFF hydrophobic module.

### Diverse molecular restructuring of the most distal iCtr maintains high TRESK activity

The complex regulation of the TREK and TASK channels was reported to rely, in part, on the altered association of the proximal region of their long iCtr to the plasma membrane (40, 41, 42, 43). Although the sequence of TRESK iCtr is not similar to these structures, the importance of the hydrophobic residues urged us to examine whether the interaction of TRESK iCtr with the plasma membrane has functional significance. We replaced the distal iCtr following the VMLFF motif with different amino acid sequences. Since poly-lysine is generally known to interact with the negative inner surface of the plasma membrane, we tested whether a chain of 12 consecutive lysines can induce high K^+^ channel activity (see the 12K construct in Table 1). Trp has the highest interfacial hydrophobicity among the amino acids (39); therefore, we also designed the 4K4W2K construct containing a shorter poly-lysine sequence with four inserted tryptophans, in an attempt to augment membrane-association also by hydrophobic interactions. In two further constructs, we applied sequences, which were expected to interact less with the membrane, a hydrophilic chain of 12 asparagines in the 12N, and the negatively charged repeat of twelve aspartates in the 12D construct (Table 1).

**Figure 8:**
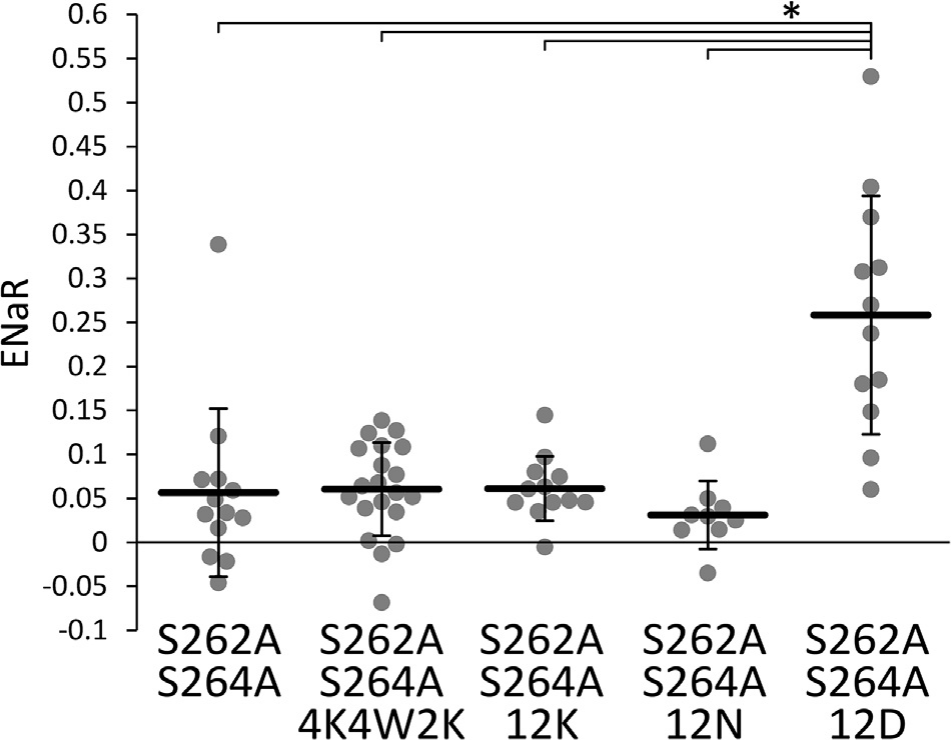
**Diverse modifications of the most distal iCtr are compatible with high TRESK activity.** The most distal iCtr of S262A + S264A double mutant ENaCα-TRESK was replaced with 12 lysines (*12K*), asparagines (*12N*), aspartates (*12D*), or a shorter combination of lysines and tryptophans (*4K4W2K*). ENaR values of these constructs, and the positive control S262A + S264A channel, were plotted, as indicated *below the graph*. The method of measurement was the same as in Figure 2. ∗*p* < 2 × 10^−4^, one-way ANOVA, Tukey HSD test (n = 13, 20, 12, 9, and 12, respectively). ENaR, epithelial sodium current ratio; iCtr, intracellular C-terminal region

The above C-terminal modifications were tested in the S262,264A constitutively active mutant. The ENaRs of the 12K and 4K4W2K constructs were practically identical to the S262,264A positive control channel (Fig. 8), suggesting that these motifs functionally substituted for the wild-type iCtr. The average ENaR of the 12N construct was lower than that of the S262,264A control, but the difference was not statistically significant. However, as an unexpected result, the 12D construct produced significantly higher ENaR values than the other groups in this experiment. In summary, the motifs (12K, 4K4W2K) containing positive and/or hydrophobic residues (like the native iCtr following the VMLFF sequence) can maintain high channel activity. However, the presence of positive and/or hydrophobic residues in this region is not absolutely necessary, as exemplified by the 12D construct.

### Substitution of the most distal part of TRESK iCtr with the membrane-anchoring motif of K-Ras 4B effectively increases K^+^ channel activity

In order to further examine the possible effects of the interaction of the iCtr with the plasma membrane, the above-described deletion mutants were extended with the membrane-anchoring motif of K-Ras 4B (see the 356-, 367-, and 375-6Kcaax sequences in Table 1). This motif includes the polybasic sequence of six lysines and the CAAX motif CVIM (44, 45, 46). The CAAX motif of Ras is farnesylated and functional in *Xenopus* oocytes (47, 48).

**Figure 9:**
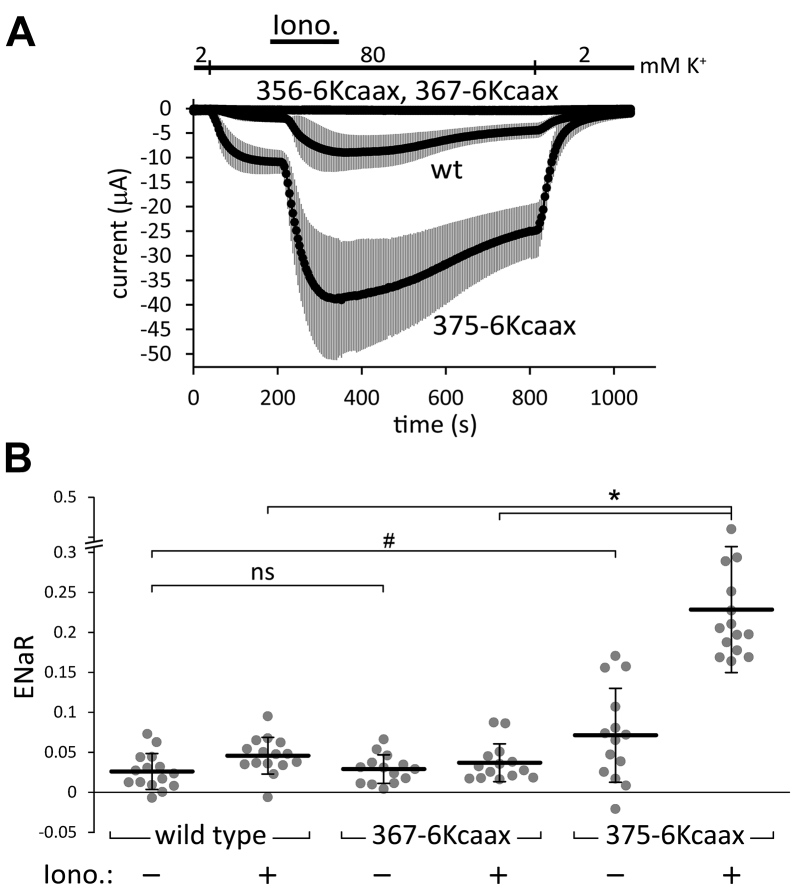
**Effects of the fusion of K-Ras 4B membrane-anchoring motif with TRESK iCtr on the K**^**+**^**currents and ENaR values, before and after ionomycin.***A*, conventional TEVC measurement of four groups of oocytes expressing wild type (*wt*), *356-6Kcaax*, *367-6Kcaax*, or *375-6Kcaax* TRESK, (n = 10, 6, 6, and 12), respectively. Average currents ± S.D. are plotted. The cells were stimulated with ionomycin (*Iono.*, 0.5 μM), as indicated by the *black bar*. The 356-6Kcaax and 367-6Kcaax channels are not expressed and these curves overlap. *B*, ENaR analysis of the 6Kcaax modifications in ENaCα-TRESK (see the groups *below the panel*). The method of measurement was the same as in Figure 2; however, the Ba^2+^-sensitive K^+^ current was measured twice in each cell, before (*Iono. −*) and after the application of ionomycin (*Iono. +*, 0.5 μM, as indicated *below the graph*). ∗*p* < 2 × 10^−4^, ^#^*p* = 0.068, ns: not significant, two-way mixed-design ANOVA, Tukey HSD test (n = 15, 14, and 14, respectively). (The *375-6Kcaax Iono +* group is different from all the other groups at *p* < 2 × 10^−4^, whereas the effect of ionomycin in the wild-type group was not significant in this experiment). ENaC, epithelial Na+ channel; ENaR, epithelial sodium current ratio; iCtr, intracellular C-terminal region; TRESK, Twik-Related Spinal cord K+ channel, K2P18.1, KCNK18.

The 356- and 367-6Kcaax constructs could not be expressed; however, TRESK with the 375-6Kcaax modification resulted in larger K^+^ currents than the wild-type channel in conventional TEVC measurements (Fig. 9*A*). We prepared the 367-6Kcaax and 375-6Kcaax versions of the ENaCα-TRESK fusion construct, and compared their average ENaR values to those of the wild-type control, before and after the application of ionomycin (Fig. 9*B*). The ENaRs of 367-6Kcaax were not significantly different from the wild type either before or after ionomycin. The basal ENaRs of 375-6Kcaax showed a tendency to be higher than those of the wild-type channel before ionomycin (0.071 ± 0.059 (n = 14) *versus* 0.026 ± 0.022 (n = 15), *p* = 0.068, two-way repeated measures ANOVA, followed by Tukey HSD test). In addition, the ENaRs of 375-6Kcaax after ionomycin (0.228 ± 0.079, n = 14) were significantly higher than those in the wild-type (0.045 ± 0.023, n = 15, *p* < 0.005) or 367-6Kcaax groups (0.037 ± 0.024, n = 14, *p* < 0.005, Fig. 9*B*). Thus, the K-Ras membrane-anchoring motif functionally substituted for the most distal part of the TRESK iCtr, when it was positioned after the maintained VMLFF sequence. The K-Ras motif was even more effective in the generation of high channel activity than the wild-type ending of TRESK iCtr.

### The fusion with TRESK iCtr prevents the cytoplasmic localization of the green fluorescent protein

**Figure 10:**
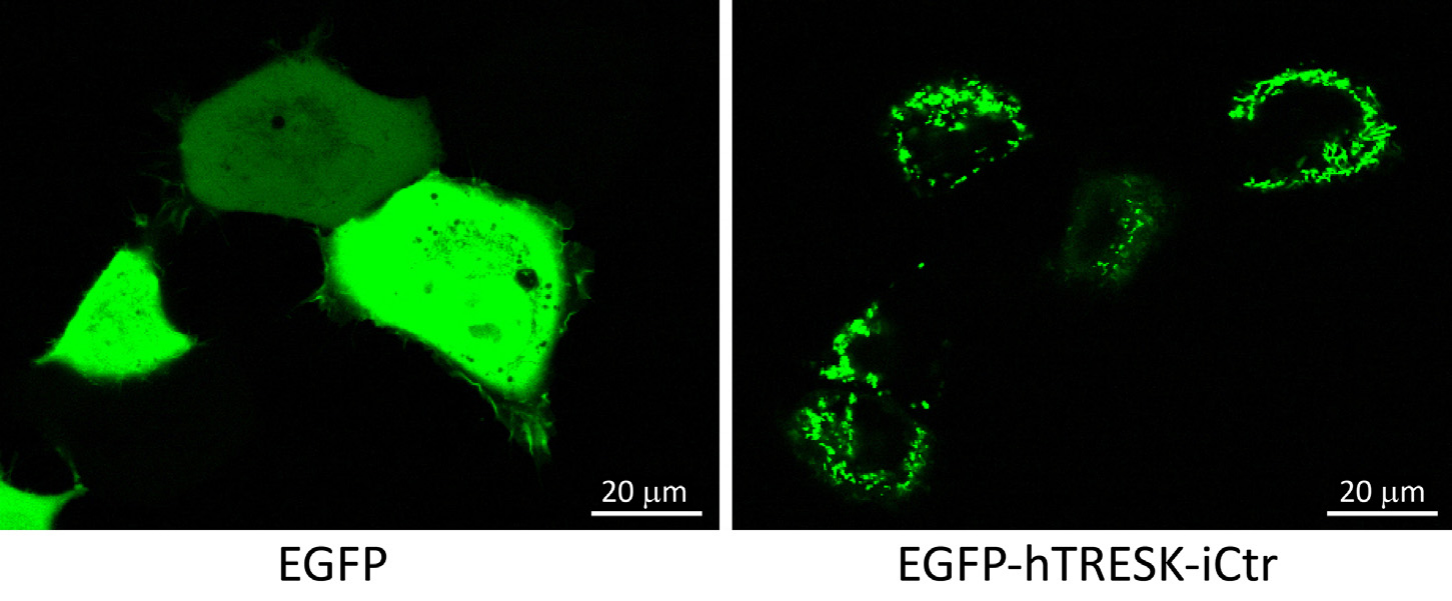
**Confocal micrograph showing the localization of EGFP fused to TRESK iCtr.** Confocal microscope images of HEK293A cells expressing the enhanced green fluorescent protein (*EGFP*, *left panel*), or the *EGFP-hTRESK-iCtr* construct (*right panel*). The addition of the 29 amino acids of TRESK iCtr to the C-terminus of EGFP prevents uniform cytoplasmic localization. The magnification is the same on both panels. For a more extensive set of images, see Fig. S7. EGFP, enhanced green fluorescent protein; ENaC, epithelial Na+ channel; ENaR, epithelial sodium current ratio; iCtr, intracellular C-terminal region; TRESK, Twik-Related Spinal cord K+ channel, K2P18.1, KCNK18.

The iCtr of human TRESK was connected to the C-terminus of EGFP, the enhanced green fluorescent protein. This fusion construct was expressed in human embryonal kidney (HEK-293A) cells, and its localization was compared to the control EGFP by confocal microscopy. The addition of the 29 amino acids of iCtr substantially changed the localization. In contrast to the typical cytoplasmic distribution of EGFP, the EGFP-hTRESK-iCtr fusion protein was almost completely absent in the cytoplasm, and it mainly appeared as a bright fluorescence associated to intracellular structures (Fig. 10). In a few dividing or closely apposed cells, a faint plasma membrane localization of EGFP-hTRESK-iCtr could also be present (Fig. S7, *white arrows* in panels 3 and 5 of the EGFP-hTRESK-iCtr group); however, this has not been further pursued, since the majority of fluorescence was distant form the plasma membrane. Because the TRESK channel, the current of which is investigated in this study, is localized in the plasma membrane, its iCtr cannot reach the deep intracellular structures. Therefore, we did not examine further, which intracellular organelle shows the highest affinity for TRESK iCtr binding. Nevertheless, the result of this experiment clearly indicates that the iCtr has the property of being sequestered from the cytoplasm.

### Unprecedented ENaR levels are reached by the replacement of the iCtr with the RW361 sequence designed to interact with the inner surface of the plasma membrane

We have practically rebuilt the distal iCtr by taking advantage of the above results, in order to engineer a variant of human TRESK displaying higher activity than the wild-type version. We applied the principle of RL and RW peptides, which have been extensively characterized in respect of their interaction with the surface of the phospholipid bilayer (49, 50, 51, 52). Since KLVQNR, the proximal six amino acid residues of the iCtr, seemed to be related to gating modulation, this region was left unchanged. The following residues were replaced with arginines and leucines, on the one hand, to follow the hydrophobicity pattern of native iCtr sequence, and on the other hand to correspond to a fragment of the RL16 peptide (52). The native VMLFF motif was replaced with LLLWW, since leucine is more hydrophobic than valine and methionine, and tryptophan has higher interfacial hydrophobicity than phenylalanine (39, 53). Finally, the most distal part of the iCtr, which is relatively tolerant, was replaced with a series of six lysines connected through a flexible glycine. We call the resulting sequence RW361 (see Table 1).

**Figure 11:**
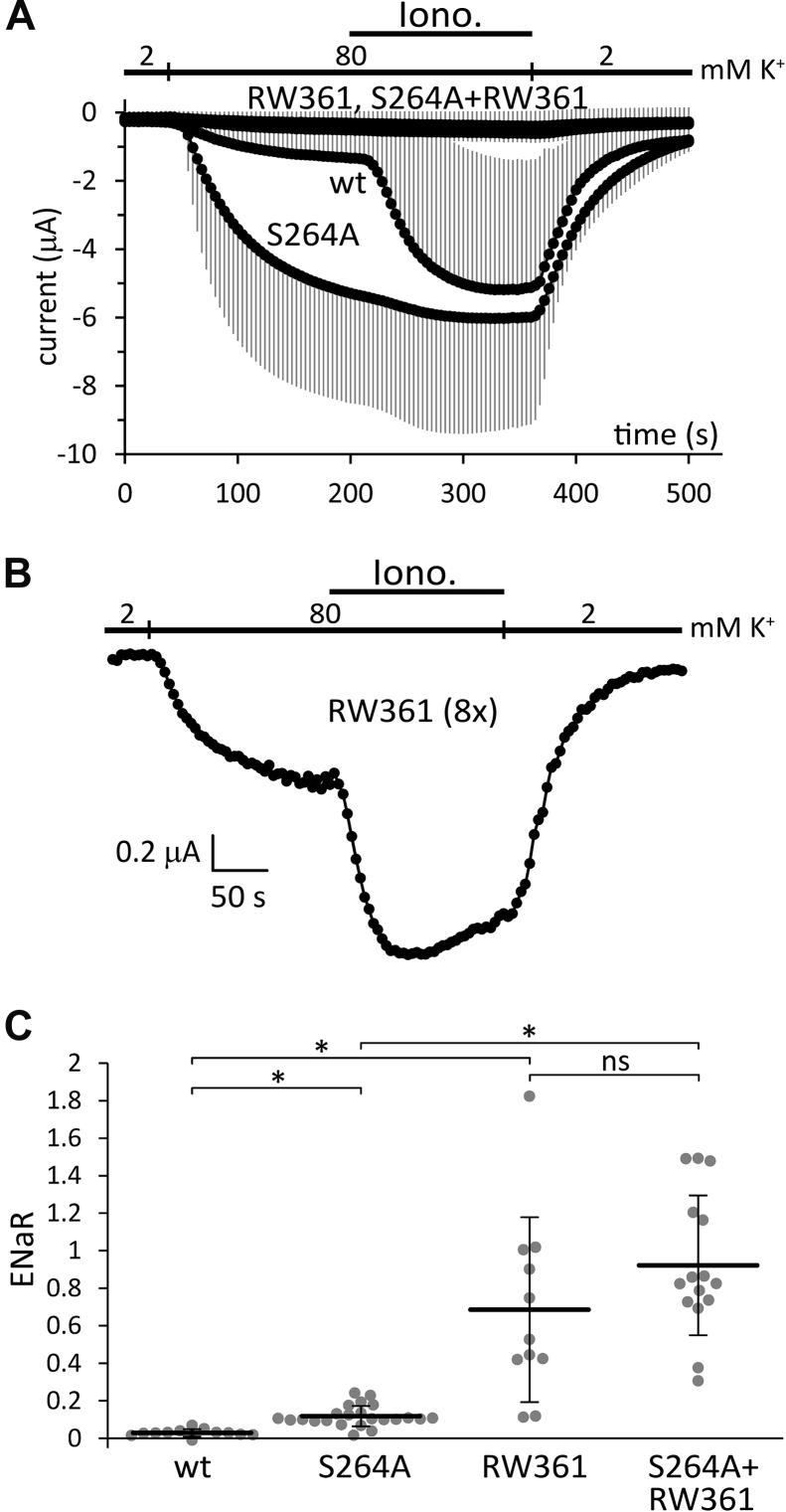
**RW361 modification of TRESK iCtr results in extraordinarily high ENaR values and decreases channel expression.***A*, conventional TEVC measurement of four groups of oocytes expressing wild type (*wt*), *S264A*, *RW361*, or *S264A* + *RW361* TRESK constructs (n = 12, 11, 5, and 12, respectively). Protocol was the same as in Figure 4*A*. Only small K^+^ currents were induced by the expression of RW361 and S264A + RW361 constructs; these *curves* partially overlap. *B*, Representative current recording from an oocyte microinjected with eight times (*8×*) higher amount of cRNA of the RW361 construct than in panel *A*. This cell evidently expressed TRESK current, which was activated in response to ionomycin (*Iono.*, 0.5 μM, *black bar*), although the degree of activation could not be calculated because the K^+^ current was in the range of endogenous K^+^ conductance of the oocyte (see the *vertical scale bar*). *C*, ENaR analysis of the wild type (*wt*), *S264A*, *RW361*, and *S264A* + *RW361* ENaCα-TRESK constructs, as indicated *below the graph*. The method of measurement was the same as in Figure 2. The reorganization of the distal iCtr by the RW361 modification resulted in remarkably high ENaR values in the RW361 and S264A + RW361 groups (note the *vertical scale*). ∗*p* < 2 × 10^−4^, ns: not significant, one-way ANOVA after logarithmic transformation, Tukey HSD test (n = 12, 23, 11, and 15, respectively). ENaC, epithelial Na+ channel; ENaR, epithelial sodium current ratio; iCtr, intracellular C-terminal region; TRESK, Twik-Related Spinal cord K+ channel, K2P18.1, KCNK18.

The iCtr was modified to RW361 in both the wild type and S264A mutant TRESK channels. These RW361 and S264A + RW361 constructs induced very small K^+^ currents, compared to the control wild-type and S264A channels, in conventional TEVC measurements (Fig. 11*A*). If the microinjected amount of RW361 cRNA was increased eight-fold, then the activation of the K^+^ current by ionomycin could be detected in the cells showing the highest expression levels (Fig. 11*B*). Altogether, the conventional whole-cell electrophysiology data, on their own, would not suggest that the RW361 constructs are highly active channels.

However, we also examined the RW361 and S264A + RW361 constructs in ENaR experiments (Fig. 11*C*). These constructs produced extraordinarily high ENaR values, much higher than the S264A constitutively active TRESK (note the vertical scale in Fig. 11*C*). In fact, we have never seen so high ENaRs with any of the TRESK constructs, but this degree of activity was more similar to that measured in the case of the TASK-3 channel (Fig. S4).

### The high activity of the S264A + RW361 construct is verified at the single-channel level

At this point, the conclusion that the RW361 constructs are highly active channels was supported only by the ENaR data. Since ENaR is a newly developed method, we examined the activity of the S264A + RW361 channel also by another independent approach, by performing single-channel measurements. The S264A + RW361 construct was compared to the S264A + Δ374 deletion mutant, which was predicted to have low activity by the ENaR experiments. The single-channel measurements were performed in the cell-attached configuration, since TRESK activity may change after the patch-excision. (The modified TRESK subunits were examined without ENaC in the single-channel experiments.)

**Figure 12:**
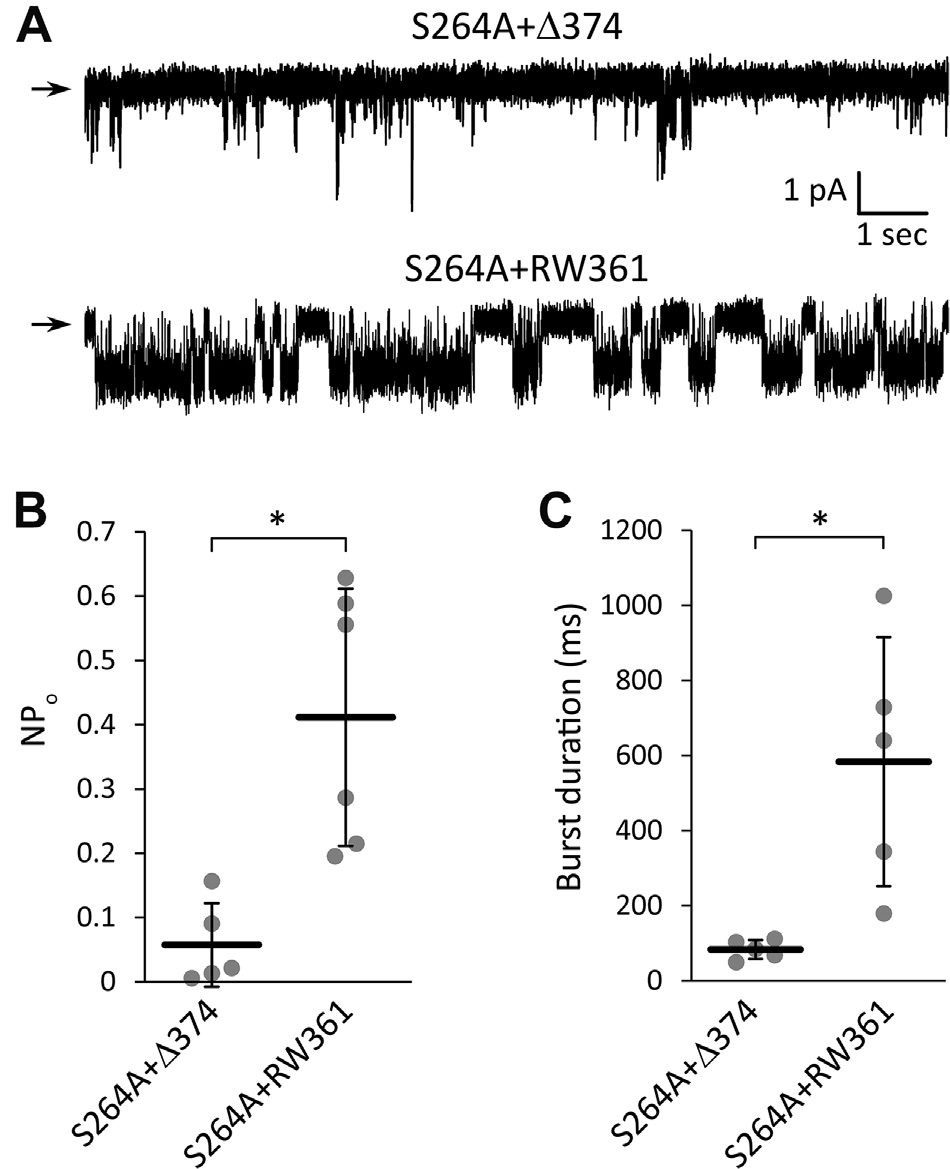
**Single-channel analysis of S264A mutant TRESK channels, truncated at amino acid 374, or modified by the replacement of the iCtr with RW361 sequence.***A*, representative single channel current recordings from two *Xenopus* oocyte membrane patches in the cell-attached configuration. In the upper recording, the patch contained *S264A + Δ374* TRESK channels (more than one), while in the lower recording, a single *S264A + RW361* TRESK channel was present. Zero current levels are indicated with *horizontal arrows*. The potential of the pipette solution was clamped to −100 mV. For more recordings, see Fig. S7. *B*, statistical analysis of the NP_o_ values of *S264A + Δ374* and *S264A + RW361* TRESK channels, as indicated *below the graph*. The *grey circles* represent the average NP_o_ from different membrane patches. ∗*p* < 0.01, Student’s *t* test (n = 5, and 6, respectively). *C*, statistical analysis of the burst duration of *S264A + Δ374* and *S264A + RW361* TRESK channels, as indicated *below the graph*. The *gray circles* represent the average burst duration from different membrane patches. ∗*p* < 0.03, Student’s *t* test (n = 5 in both groups). iCtr, intracellular C-terminal region; TRESK, Twik-Related Spinal cord K+ channel.

The S264A + RW361 channel showed apparently higher probability of the open state (P_o_) than the S264A + Δ374 deletion mutant (see Fig. 12*A* for representative recordings; see Fig. S8 for a more extensive dataset). Because of the high P_o_ of the S264A + RW361 construct, the number of channels in the patch could be determined from the simultaneous openings. In this way, we selected the membrane patches containing only one S264A + RW361 mutant TRESK channel (or in an exceptional case, two channels, Fig. S8). Thus, we could plot real P_o_ values for the S264A + RW361 construct in Figure 12*B*. However, the number of channels in the membrane patch could not be determined for the low activity S264A + Δ374 mutant, because the maximum level of simultaneous openings underestimates the actual number of channels. Therefore, NP_o_ values are given for the S264A + Δ374 construct, which obviously overestimates the real P_o_, since an unknown number of channels could be present in the analyzed patches. Despite this evident limitation of the single-channel analysis, which generally prevents the exact comparison of low channel activities, even the P_o_ values of the S264A + RW361 construct were significantly higher than the NP_o_ values of the S264A + Δ374 mutant (Fig. 12*B*).

TRESK open state is frequently interrupted by short closures in the low millisecond range. Therefore, we defined the burst of channel activity as the continuous series of open states not separated by a closed state longer than 50 ms. This calculation may overestimate the real burst duration of the S264A + Δ374 construct, because of the sequential random openings of different channels in the patch. Still, the burst duration of the S264A + RW361 construct, calculated in this way, was significantly longer than that of the S264A + Δ374 mutant (Fig. 12*C*).

In summary, the single-channel analysis confirmed that the S264A + RW361 channel is more active than the S264A + Δ374 construct, in good agreement with the conclusion derived from the ENaR data. The polypeptide sequences of native proteins are streamlined to perform multiple functions; the TRESK iCtr is a major positive determinant of channel activity and is also important for the expression in the plasma membrane. The low expression level of the RW361 constructs limited the current amplitudes in the conventional TEVC measurements. However, the channel activity was remarkably increased by the RW361 replacement, as indicated by the ENaR and single-channel experiments.

## Discussion

The resting membrane potential and cellular excitability are regulated by K_2P_ channel activity and the consequent background K^+^ conductance of the plasma membrane in a wide variety of cell types (5). In accordance with the prevailing theory of K_2P_ channel regulation, the diverse modulatory effects converge on the long C-terminal tail and influence the gating of the pore *via* the conformational changes of the fourth transmembrane segment, TMS4 (54, 55, 56, 57), or the X-gate (58, 59). This model was mainly developed based on the most intensively examined TREK-1 and TASK-1 channels, although it was also suggested to apply to the other members of the K_2P_ family. In the case of TREK-1, the intracellular pH, membrane stretch, temperature, polyunsaturated fatty acids, phosphorylation by protein kinases, and interacting proteins influence K^+^ channel activity *via* the C-terminal region (for a short summary, see ref. (60)).

In contrast to the other K_2P_ channels, the currently known regulatory mechanisms of TRESK rely on the long intracellular loop between the second and the third TMSs (Fig. 1). In the past 2 decades, we identified the structural determinants of TRESK regulation by calcineurin, 14-3-3 adapter protein, microtubule-affinity-regulating kinase (MARK), protein kinase A (PKA), and novel-type protein kinase C (nPKC) in the intracellular loop (for reviews, see (1, 2)). However, at present, to our knowledge, no physiological regulatory mechanism is known to act *via* the exceptionally short intracellular C-terminal region (iCtr) of TRESK. Before the present study, the function of TRESK iCtr could be considered uncharted territory.

In order to examine the channel activity of the TRESK constructs modified in the iCtr, we developed the Epithelial Na^+^ current Ratio (ENaR) method. The ENaR value is calculated as the K^+^ current divided by the Na^+^ current, both conducted by the protein complex containing the interconnected TRESK and ENaC channels. This ENaR value is proportional to i_K_P_oK_ in single channel terms, that is, ENaR = αi_K_P_oK_, where i_K_ is the unitary conductance, P_oK_ is the probability of the open state of the K^+^ channel, and α is a constant, which may vary in the different cell preparations and depend on the experimental conditions (For the simple derivation of the equation, see Fig. S9 in the Supporting information). Accordingly, i_K_ and P_oK_ are not separately quantified by the ENaR value; instead, ENaR is a parameter proportional to the average current of a single K^+^ channel. In fact, ENaR estimates the physiologically most relevant function of ion channels under whole-cell conditions.

In the present study, we report for the first time that the iCtr is a fundamental determinant of TRESK activity. Although the iCtr of TRESK is short, only 29 amino acids, its different parts are responsible for diverse functions. The mutations of positive residues in the proximal iCtr, in the KLVQNR sequence following the fourth TMS, interfered with the normal transmission of regulatory effects to the gating apparatus of TRESK. The K356E and R361E mutants operate in a sustained, low-activity mode, decoupled from the regulatory control. The most probable explanation for the effect of the K356E and R361E mutations is that the KLVQNR region directly contributes to the transmission of regulatory modulation. The K356 and R361 residues may form electrostatic interactions with the adjacent structures of the channel protein and thus transmit force, or interact with the membrane by a snorkeling mechanism at the intracellular end of TMS4 (61). Mutations of the proximal iCtr were also found to obstruct regulatory mechanisms in other K_2P_ channels (40, 41, 42, 62).

The RLIDIY hydrophobic module in the middle of the iCtr is necessary for the maintenance of high TRESK activity since the substitution of its hydrophobic residues with alanines is incompatible with the high ENaR of the channel activated by the S264A mutation. The side chain complexity of the RLIDIYKNV region was substantially changed and reduced when it was replaced with the corresponding fragment of the RL16 peptide. The altered side chain complexity would likely affect protein-protein interactions, yet the RW361 construct showed very high activity. Considering the well-characterized association of the RL16 α-helix with the interface of the phospholipid bilayer (50, 52), the overlying of the middle part of the iCtr on the cytoplasmic surface of the plasma membrane may be an essential mechanism of TRESK activity.

The substitution of the VMLFF sequence with alanines impaired the high activity of the S264A mutant TRESK. Therefore, this region of the iCtr is also required for the active state. The LLLWW motif of the RW361 construct functionally replaced the five hydrophobic amino acids of the native VMLFF region. Therefore, this region also tolerated the alteration of amino acid side chains, when the hydrophobic character was maintained (or exaggerated); even the bulky indole moieties of the tryptophans were compatible with the function. Accordingly, the data are consistent with the hypothesis that the VMLFF region of the iCtr also interacts with the membrane. The aliphatic side chains may submerge toward the hydrophobic core of the phospholipid bilayer, with the possible arrangement of the two phenylalanines (or tryptophans) at the favored location of these amino acids, the membrane interface.

For the maintenance of the high channel activity, an amino acid sequence has to follow the VMLFF region, since the Δ374 deletion decreased the ENaR of the S264A channel. However, several sequence combinations, containing hydrophilic amino acids, were appropriate substitutions for the most distal iCtr. In addition to the wild-type sequence, the 375-6Kcaax, 4K4W2K, 12K, and 12D designs also contained hydrophilic amino acids in the vicinity of the VMLFF motif, and maintained or even increased the channel activity of the S264A (or S262,264A) construct. These hydrophilic residues may not partition in the hydrophobic phase of the plasma membrane, although the sequence variants containing positive amino acids, as in the wild-type sequence, may interact with the head groups of acidic phospholipids. We envision that the VMLFF and the following hydrophilic region constitute a structure similar to a buoy, which is fixed (or oscillates around a point) in a given distance from the midplane of the phospholipid bilayer. This buoy connected to the rigid helical middle part of the iCtr may act like a lever, and propagate the movements efficiently to the proximal iCtr for the modulation of gating.

In the present study, we have taken the first steps toward the elucidation of the function of TRESK iCtr. The application of the ENaR method has proved to be an excellent tool for the functional analysis of permanent sequence modifications, which would have been difficult to perform by using the conventional methods of electrophysiology. We have reached two definite conclusions. First, the native iCtr contributes to the maintenance of high TRESK channel activity. Second, outstanding TRESK activity can be achieved by an appropriate modification of the iCtr. These results are novel and unexpected in the sense that no previous data suggested the importance of TRESK iCtr, and the obtained functional effects are remarkably robust compared to the calcineurin-dependent regulation of the channel. On the other hand, the data correspond well to the overall importance of the iCtr in the K_2P_ channel family, and the short iCtr of TRESK may be a suitable model for a better understanding of K_2P_ gating modulation in general. In addition to the two major conclusions, the ENaR measurements provided a wide array of data about the functional landscape of the iCtr, and we offered parsimonious explanations for the results in this discussion. Evidently, further investigation is required to establish whether the present interpretation of the functional data is tenable or whether the underlying mechanisms turn out to be more complex.

## Experimental procedures

### Materials

Chemicals of analytical grade were purchased from Sigma, Fluka, Enzo Life Sciences, Santa Cruz Biotechnology, or Merck. Enzymes and kits for molecular biology applications were purchased from Ambion, QIAGEN, Thermo Fisher Scientific, New England Biolabs, and Stratagene.

Ionomycin (calcium salt, Enzo Life Sciences) was dissolved in dimethyl sulfoxide (DMSO) as 5 mM stock solution. Amiloride (Sigma) and A2793 (36) were dissolved at 50 mM concentration in DMSO. Cloxyquin (5-Chloro-8-quinolinol, Sigma) was dissolved in ethanol (100 mM), and carbachol in water (10 mM). The stock solutions were stored at −20 °C, and diluted in the high [K^+^] solution before the application.

### Molecular biology

Plasmid constructs were produced with standard PCR and cDNA cloning techniques. Phusion polymerase and most restriction enzymes were purchased from Thermo Fisher Scientific. *In vitro* site-directed mutagenesis was performed according to the manufacturer's instructions of the QuikChange site-directed mutagenesis kit (Stratagene). Extended sequence modifications were assembled by the application of overlapping oligonucleotides.

The coding sequence (CDS) of the N-terminal 624 amino acids of ENaC α subunit (GeneBank accession number: AAI33689.1) was amplified by RT-PCR (RevertAid Reverse Transcriptase, Thermo Fisher Scientific) from mouse kidney total RNA purified with TRIzol reagent (Invitrogen, Carlsbad, CA). The CDS of the N-terminal 563 amino acids of ENaC β (GeneBank: NP_001258952.1), and 575 amino acids of ENaC γ (GeneBank: NP_035456.1), were similarly amplified. The cloning of human TRESK (GeneBank: NP_862823.1) was previously described (10). In the ENaCα-TRESK fusion constructs, the C-terminus of ENaC α subunit was connected to TRESK without a linker, *via* the …RYWSPGSGHPQAR… amino acid sequence, where the TRESK residues are underlined, but the ENaC α is not. The joining sequence for human TASK-1 and mouse TASK-3 was …RYWSPGSGKRQNVRT…, where the identical N-terminal residues of the two TASK subunits are underlined. These constructs were cloned into the pXEN vector (GenBank: EU267939.1).

The iCtr of human TRESK was modified in the TRESK-pXEN plasmids for the conventional TEVC measurements and in the ENaCα-TRESK-pXEN plasmids for the epithelial sodium current ratio (ENaR) experiments. The modifications are summarized in Table 1.

The EGFP-hTRESK-iCtr construct was used for confocal microscopy. The iCtr of human TRESK was fused to the C-terminus of enhanced green fluorescent protein (EGFP) in the pEGFP-C1 vector (Clontech). The joining sequence was …RSRAQASNSGGKLVQNR…, where the proximal iCtr of TRESK is underlined. The CDS of all constructs was verified by Sanger sequencing (Microsynth AG).

The cRNAs of the different TRESK and ENaCα-TRESK constructs were synthesized using the mMESSAGE mMACHINE T7 *in vitro* transcription kit (Ambion), according to the instructions of the manufacturer. The plasmid templates for the reaction were linearized with XbaI, NotI, or SmaI restriction enzymes. The quality of all cRNA preparations was verified by denaturing formaldehyde agarose gel electrophoresis and ethidium bromide staining.

### *Xenopus laevis* oocyte microinjection and maintenance

*Xenopus* oocytes were prepared and microinjected as previously described (29, 63). The truncated ENaC β and γ subunits were expressed very efficiently when 0.25 ng quantities of their cRNAs were microinjected per oocyte in 50 nl volume with Nanoliter Injector (World Precision Instruments). Together with the truncated ENaC α subunit (also 0.25 ng/50 nl), this triple coexpression resulted in large (>20 μA) Na^+^ currents (Fig. S2), suggesting that ENaC β and γ subunits were applied in saturating quantities in the ENaR experiments. The ENaCα-TRESK constructs had to be microinjected in 2.5 to 10 ng/50 nl quantity in order to obtain a typical 5 to 20 μA Na^+^ current, and the coinjection of ENaC β and γ cRNAs in higher amounts than 0.25 ng/50 nl did not further increase the current. The application of saturating amounts of truncated ENaC β and γ was a reasonable approach since the coexpression of ENaC β and γ without α subunit did not result in Na^+^ current (Fig. S2).

During the 3-day expression period of the TRESK-pXEN constructs, the oocytes were incubated in modified Barth’s solution, containing (in mM): 88 NaCl, 1 KCl, 2.4 NaHCO_3_, 0.82 MgSO_4_, 0.33 Ca(NO_3_)_2_, 0.41 CaCl_2_, 20 HEPES buffered to pH 7.5 with NaOH and supplemented with penicillin (100 units/ml), streptomycin (100 μg/ml), sodium pyruvate (4.5 mM), and theophylline (0.5 mM). This standard solution was appropriate for the maintenance of the cells expressing the K^+^ channel constructs. However, the oocytes coexpressing the ENaCα-TRESK constructs with ENaC β and γ did not survive in modified Barth’s solution, because of the high permeability of the plasma membrane to both Na^+^ and K^+^. In order to overcome this problem, we formulated a solution of low [Na^+^] and high [K^+^], and thereby shifted the Na^+^ and K^+^ equilibrium potentials toward the normal resting membrane potential of non-injected oocytes (≈−30 mV). This solution, called ELEN for low [Na^+^], contained (in mM): 2 NaCl, 25 KCl, 58 N-methyl-D-glucamine (NMDG), 2.4 NaHCO_3_, 0.82 MgSO_4_, 0.33 Ca(NO_3_)_2_, 0.41 CaCl_2_, 20 HEPES, buffered to pH 7.5 with HCl and supplemented with penicillin (100 units/ml), streptomycin (100 μg/ml), and theophylline (0.5 mM). The oocytes functionally expressing ENaC-TRESK constructs survived very well in ELEN solution. (Non-injected oocytes did not tolerate ELEN well).

All experimental procedures using animals were conducted in accordance with local state laws and institutional regulations. All animal experiments were approved by the Animal Care and Ethics Committee of Semmelweis University (approval ID: XIV-I-001/2154-4).

### TEVC and ENaR measurements

TEVC measurements were performed 3 days after the microinjection of cRNA, as previously described (10). In the conventional TEVC experiments, we applied two solutions, indicated as 2 or 80 mM K^+^ in the figures. (K^+^ current measurements of TRESK constructs were called as "conventional" TEVC, in order to distinguish these from the ENaR experiments.) Low [K^+^] solution contained (in mM): 95 NaCl, 2 KCl, 1.8 CaCl_2_, 5 HEPES (pH 7.5 with NaOH). High [K^+^] solution contained 80 mM K^+^ (78 mM Na^+^ of the low [K^+^] solution was replaced with K^+^). Background K^+^ currents were measured in the high [K^+^] solution at the end of 300-ms voltage steps to −100 mV applied in every 4 s.

In the ENaR experiments, Na^+^ and K^+^ currents were measured consecutively by two-electrode voltage clamp in the same oocyte coexpressing an ENaCα-TRESK construct with truncated ENaC β and γ. At the start of the ENaR measurements, the oocytes were placed in St solution, which is characterized by similar ionic composition to ELEN incubating medium. St solution contained (in mM): 4 NaCl, 25 KCl, 68 NMDG, 1.8 CaCl_2_, 5 HEPES (pH 7.5 with HCl). In the figures, St solution is not indicated with a label at the beginning of the recording. An unknown mixture of Na^+^ and K^+^ currents is measured in St solution, accordingly, this current has not been used in the calculations.

ENaC Na^+^ currents were measured as the current component inhibited by 20 μM amiloride in "15 Na^+^" solution containing (in mM): 15 NaCl, 2 KCl, NMDG, 1.8 CaCl_2_, 5 HEPES (pH 7.5 with HCl). Amiloride inhibited the truncated ENaC channel (Fig. S2) but did not affect TRESK currents (Fig. S10). TRESK, TASK-1, and TASK-3 currents were measured as the current component inhibited by 5 mM Ba^2+^ in "97 K^+^" solution containing (in mM): 97 KCl, 1.8 CaCl_2_, 5 HEPES (pH 7.5 with NMDG). Both the Na^+^ and K^+^ currents were measured with 300-ms voltage steps to −100 mV applied every 4 s. In some experiments, in addition to the control "15 Na^+^" solution with amiloride, the non-specific leak current of the oocyte was also estimated in "95 NMDG" solution containing (in mM): 95 NMDG, 2 KCl, 1.8 CaCl2, 5 HEPES (pH 7.5 with HCl).

### Single-channel recordings of TRESK activity in the cell-attached configuration

The vitelline membrane of *Xenopus* oocytes expressing the S264A + Δ374 or S264A + RW361 construct was removed with fine forceps in a hyperosmotic solution containing (in mM): 200 DL-aspartic acid, 20 KCl, 1 MgCl_2_, 5 ethylene glycol-bis(2-aminoethylether)-N,N,N′,N′-tetraacetic acid (EGTA), 10 HEPES (pH 7.4 with KOH). Single channel activity was recorded in the cell-attached configuration with RK-400 amplifier (Biologic, Claix, France) using microelectrodes made of borosilicate glass (World Precision Instruments, BF120-69-10) with a resistance of 50 to 150 MΩ when fire-polished and filled with pipette solution. The pipette solution contained (in mM): 140 KCl, 4 MgCl_2_, 1 CaCl_2_, 10 HEPES (pH 7.4 with NaOH). The bath solution was similar to the pipette solution but contained 5 mM EGTA instead of CaCl_2_. Single channel currents were recorded at about +100 mV (cytoplasmic side positive, *i.e.*, the potential of the pipette solution was clamped at −100 mV). Seal resistance was at least 20 GΩ. The currents were low-pass filtered at 0.3 kHz and digitally sampled at 1 kHz by Digidata Interface 1440A (Molecular Devices). Data were analyzed by pCLAMP Clampfit 11 (Molecular Devices). The acquisition of single-channel data has been performed for weeks, on a trial-and-error basis. A high number of recordings were omitted from the analysis, because these contained multiple channels, no channels at all, or were contaminated with SAC, the 27 pS stretch-activated channel (64) or other endogenous channel activity of the oocyte, instead of the 13 to 16 pS TRESK single channel conductance (10).

### Analysis of TRESK iCtr localization by confocal microscopy

Human embryonal kidney (HEK) 293A cells (R705-07, Thermo Fisher Scientific) were transfected with 0.1 μg EGFP-hTRESK-iCtr or pEGFP-C1 control plasmid and 0.5 μl Invitrogen Lipofectamine 2000 reagent (Thermo Fisher Scientific) in 440 μl DMEM without FCS (Dulbecco′s Modified Eagle′s Medium, no Fetal Calf Serum) per well of ibidi μ-Slide 8-Well plates (ibidi Ltd) for 4 h. After the transfection, the cells were incubated in DMEM supplemented with 10% FCS, and examined next day. Living cells were imaged after replacing DMEM-FCS with a physiological salt solution containing (in mM): 138 NaCl, 4 KCl, 1 MgCl_2_, 1 CaCl_2_, 10 HEPES (pH 7.4 with NaOH). Images were captured with a Nikon A1plus, Ti2 Eclipse confocal microscope, by using the 488 nm laser for excitation, 60× oil immersion objective, and NIS-Elements software 5.11.

## Data and statistical analysis

In the ENaR experiments, Na^+^ currents reached at least 10 μA under optimal conditions. A uniform cutoff value was applied in the compared groups, the cells expressing smaller Na^+^ current than 3 or 5 μA were omitted, depending on the overall expression level in the experiment, in order to avoid large variation of the I_K_/I_Na_ ratio. In some cells, the ENaR value was negative, because the K^+^ current even increased after the addition of 5 mM Ba^2+^ in "97 K^+^" solution. Although TRESK current is not increased by Ba^2+^ and the negative values are clearly affected by experimental data variation, these points were also included in the analysis for the consistency of the data groups (and shown in the figures). These negative values carry useful information; TRESK current was close to zero in the corresponding cells.

Data are expressed as mean ± SD. In most figures, individual data points are also illustrated in *grey*. The statistical difference was considered to be significant at *p* < 0.05. Unpaired, heteroscedastic Student’s *t* test was applied for the comparison of two independent sample groups, and one-way ANOVA was followed by Tukey HSD post hoc test for multiple groups (ANOVA results are summarized in Fig. S11). In one experiment, heteroscedasticity was reduced by logarithmic transformation of the data before one-way ANOVA. In another experiment, two-way repeated measures (mixed-design) ANOVA was used according to the experimental plan. The type of the applied test is always given in the figure legends. Data were also analyzed by non-parametric methods, by Mann-Whitney U test or Kruskal-Wallis ANOVA followed by the multiple comparisons of mean ranks; *p* values from the non-parametric tests are not given in the text, however, the differences, on which the most important conclusions are based, were also significant by these tests. Statistical calculations were performed with Statistica 13.5 (TIBCO Software, Tulsa, OK) or SPSS Statistics 28.0 (IBM Corporation).

## Data availability

All data needed to evaluate the conclusions are presented in this article or in the Supporting information. Correspondence and requests for materials should be addressed to G. C. (czirjak.gabor@med.semmelweis-univ.hu).

## Supporting information

This article contains supporting information.

## Conflict of interest

The authors declare that they have no conflicts of interest with the contents of this article.
