# Supplementary material for: Critical contribution of the intracellular C-terminal region to TRESK channel activity is revealed by the epithelial Na+ current ratio method
Source: J Biol Chem. 2023 Apr 20;299(6):104737. doi: 10.1016/j.jbc.2023.104737 (PMC10206819; doi:10.1016/j.jbc.2023.104737)
Supplement: Supporting Figures S1–S11 [file mmc1.pdf]

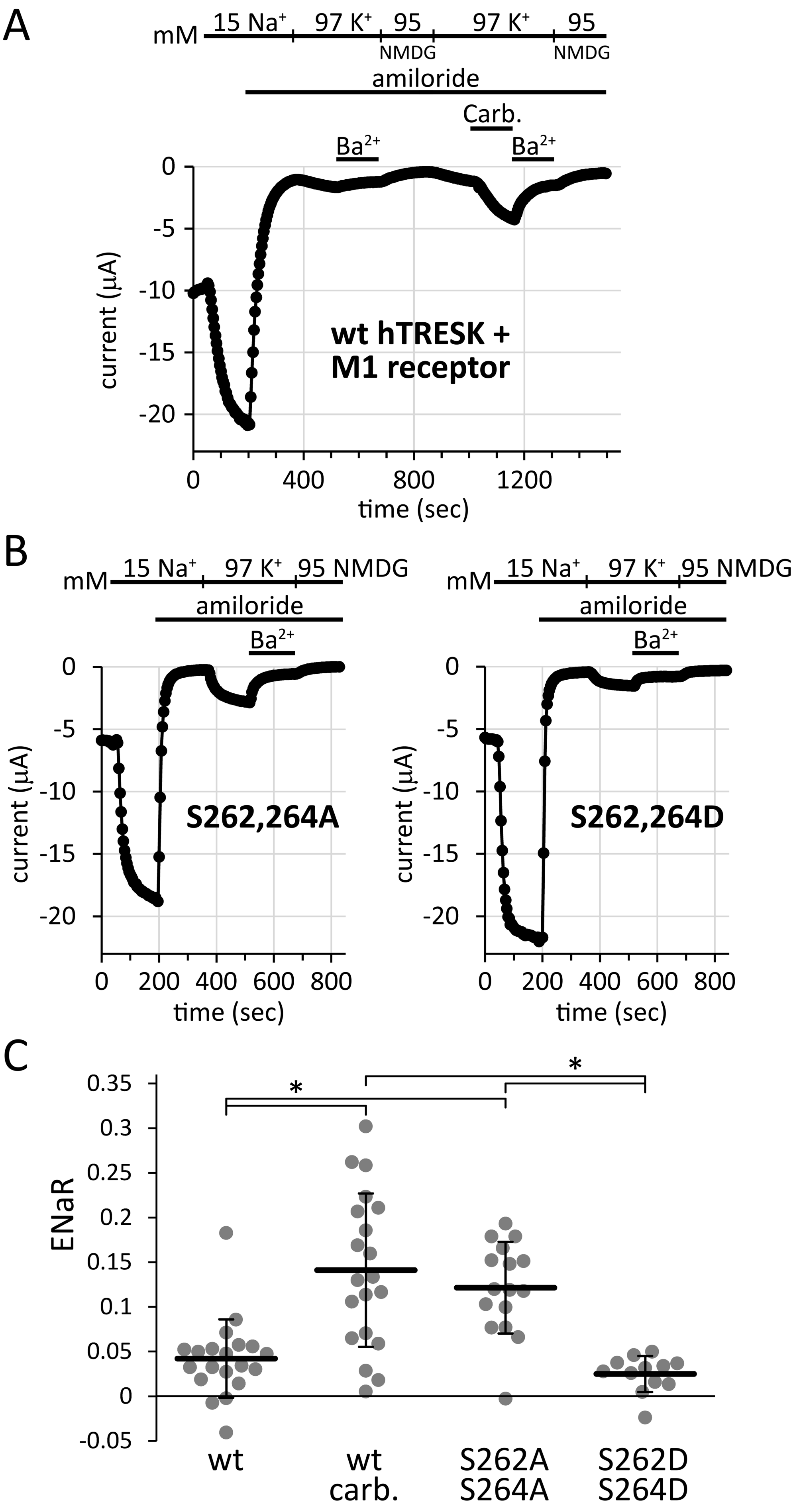

Figure S1. Regulation of ENaCα-TRESK by G_q-protein-coupled receptor stimulation.

A. Representative recording of the Na^+ and K^+ currents of a *Xenopus* oocyte coexpressing ENaCα-TRESK with the truncated ENaC β and γ, and M_1 muscarinic receptor. ENaC current was measured as the Na^+ current inhibited by *amiloride* (20 µM), whereas TRESK currents were measured twice as the K^+ currents inhibited by *Ba^{2+}* (5 mM), before and after receptor stimulation with carbachol (*Carb.*, 1 µM). As a result, two (basal and stimulated) ENaR values were assigned to each cell in this experiment.

B. Representative measurements of two oocytes coexpressing the constitutively active S262A+S264A double mutant (*S262,264A, left panel*), or the constitutively inhibited, S262D+S264D ENaCα-TRESK construct (*S262,264D, right panel*) with the truncated ENaC β and γ, in the same cell preparation as in panel A. ENaR recording protocol is the same as in Fig. 2.B of the main text.

C. Statistical analysis of the ENaR values in the three groups of oocytes expressing the constructs introduced in panels A and B (as indicated *below the graph*). The *two groups on the left* correspond to the basal and stimulated ENaR values of ENaCα-TRESK containing the wild type (*wt*) K^+ channel sequence, before and after carbachol, respectively. Each *grey circle* represents the ENaR value of a single cell. *$p < 3\times10^{-4}$, Student's t test after Bonferroni correction. The *two groups on the left* were compared by paired, while the other combinations by unpaired heteroscedastic t tests. n = 20, 16, and 12 in the *wt*, *S262,264A*, and *S262,264D* groups, respectively

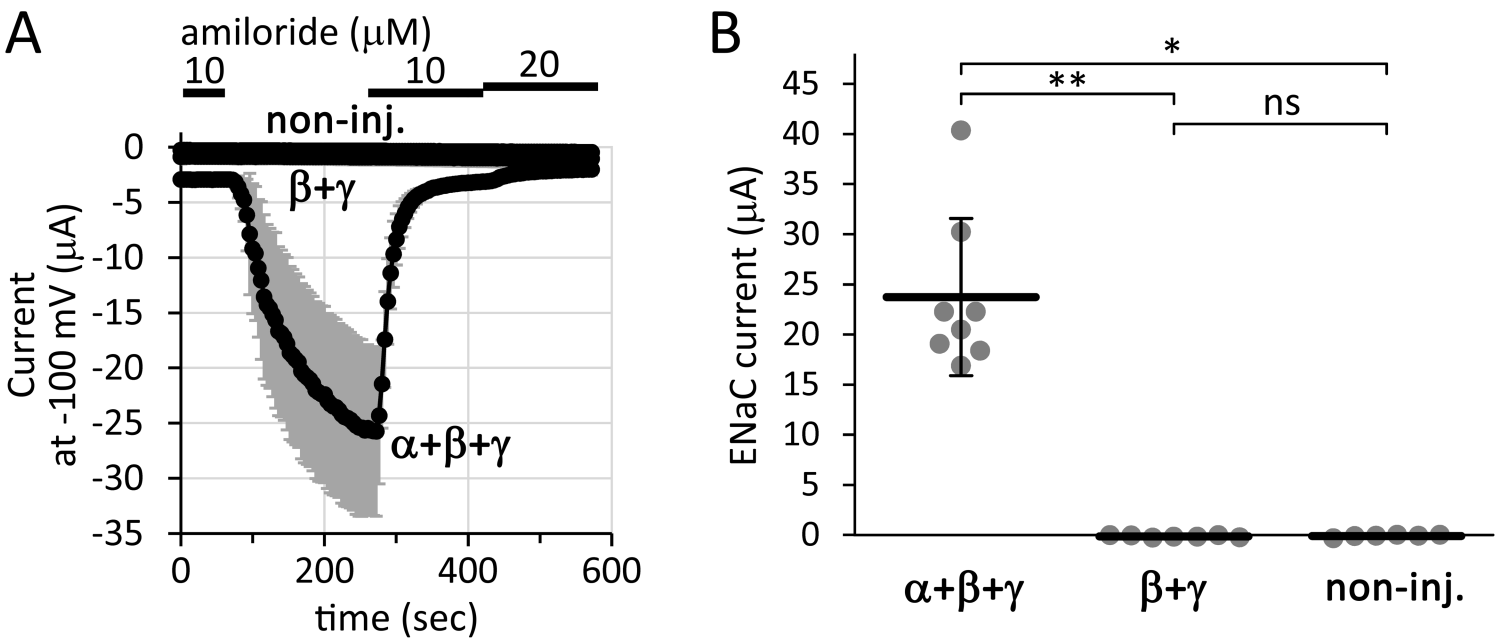

Figure S2. The α subunit is required for the functional expression of ENaC.
A. Currents in three groups of oocytes were measured by conventional TEVC, in the presence of 95 mM EC [Na^+] (low [K^+] solution; see the *Experimental procedures* in the main text). The oocytes coexpressed the truncated α, β and γ subunits of ENaC in one group (*α+β+γ*), only the β and γ subunits in another group (*β+γ*), whereas the third group was non-injected control (*non-inj.*). Amiloride (10 or 20 μM) was applied as indicated by the *horizontal black bars* above the graph. The curves of the β+γ and non-injected control groups overlap.
B. Na^+ currents inhibited by amiloride (20 μM) are plotted for the three groups introduced in panel A, and indicated *below the graph*. Each *grey circle* corresponds to one cell. *$p < 0.02$, **$p < 0.002$, ns: not significant, Kruskal-Wallis ANOVA followed by the multiple comparison of mean ranks (n = 8, 7, and 6, respectively).

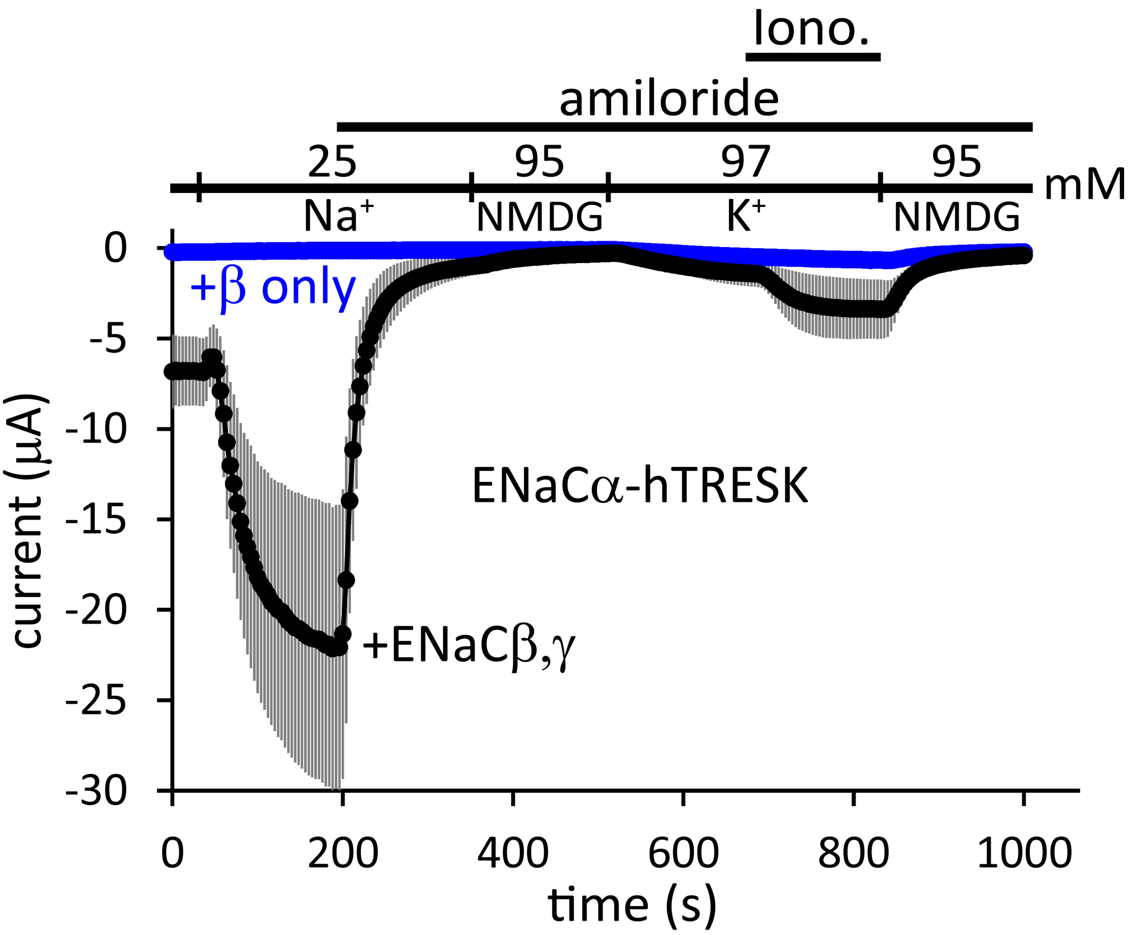

Figure S3. Coexpression of ENaCα-TRESK with only ENaC β induces neither Na^+ nor K^+ current. Average currents are plotted for two groups of oocytes coexpressing ENaCα-TRESK with only the ENaC β construct (*+β only*, n = 4, *blue curve*), or with the ENaC β and γ subunits together as a positive control (*+ENaCβ,γ*, n =19). Na^+ currents were inhibited by *amiloride* (20 μM) in 25 mM EC [Na^+] at −100 mV, K^+ currents were measured in 97 mM EC [K^+], before, and after the application of ionomycin (*Iono.*, 0.5 μM), as indicated *above the curves*. Na^+ and K^+ currents were significantly different in the two groups ($p < 4\times10^{-10}$, and $p < 10^{-6}$ (after ionomycin), respectively, Student's t test, unpaired, heteroscedastic). In further experiments, inhibition by 5 mM Ba^{2+} was applied in order to facilitate more selective detection of TRESK current.

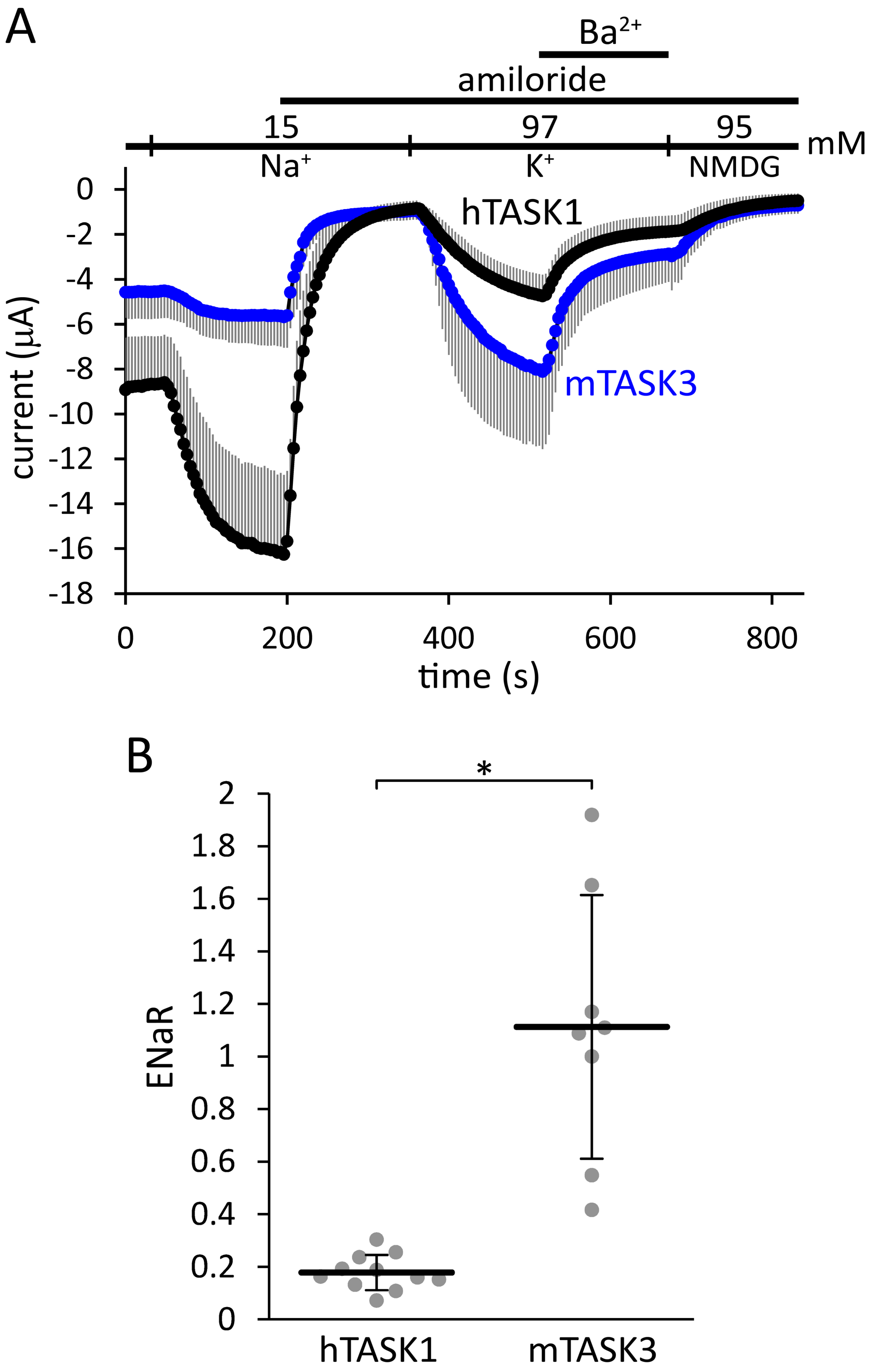

Figure S4. TASK-1 and TASK-3 channels generate high basal ENaR values.
A. Average currents are plotted for two groups of oocytes coexpressing ENaCα-hTASK1 (*hTASK1,* n = 11) or ENaCα-mTASK3 (*mTASK3*, *blue curve,* n = 8) fusion construct with the truncated ENaC β and γ subunits. Na^+ currents were inhibited by *amiloride* (20 µM) in 15 mM EC [Na^+], whereas K^+ currents by *Ba^{2+}* (5 mM) in 97 mM EC [K^+] at −100 mV, as indicated *above the curves*.
B. Statistical analysis of the experiment shown in panel A. Mouse TASK-3 induced higher ENaR values than human TASK-1 (*$p < 0.002$, Student's t test). Note the *vertical scale*. Each *grey circle* corresponds to one cell.

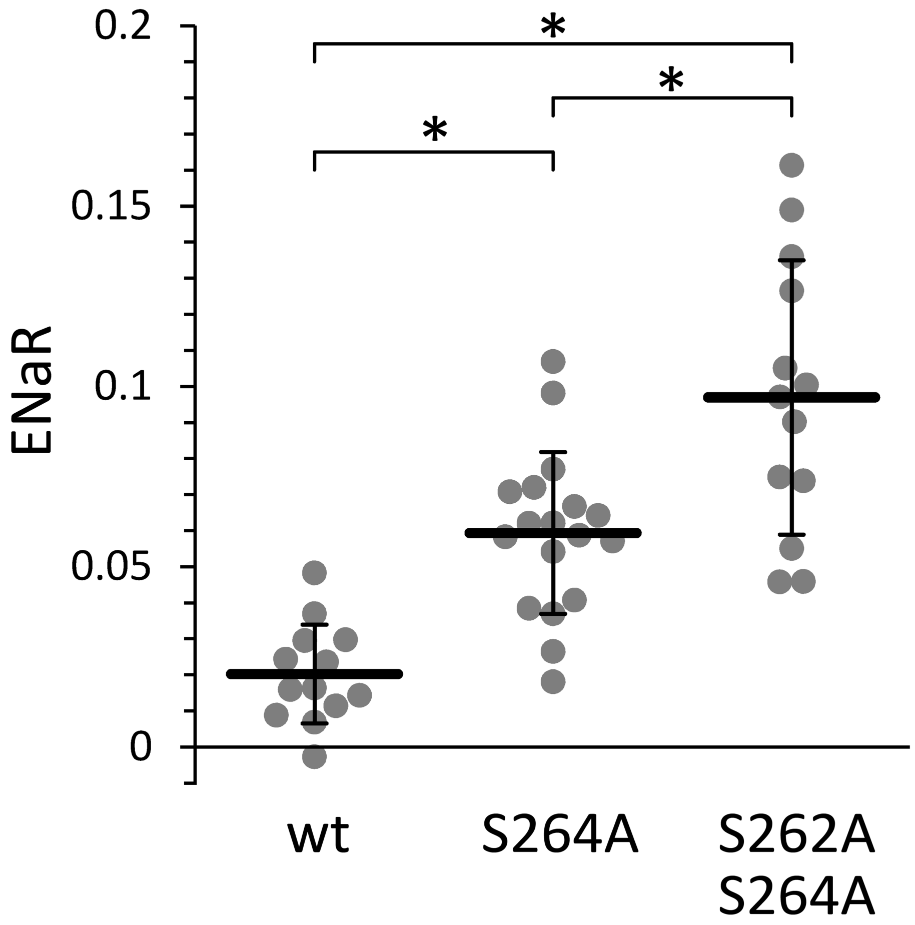

Figure S5. Comparison of the ENaR values of S264A and S262,264A constitutively active constructs to wild type TRESK.
ENaR values are plotted for three groups of oocytes expressing the wild type (*wt*), *S264A*, or *S262A+S264A* mutant ENaCα-TRESK constructs (as indicated *below the graph*). Each *grey circle* represents the ENaR value of a single cell from the same oocyte preparation. *p < 0.001, one-way ANOVA, Tukey HSD test (n = 13, 18, and 13, respectively).

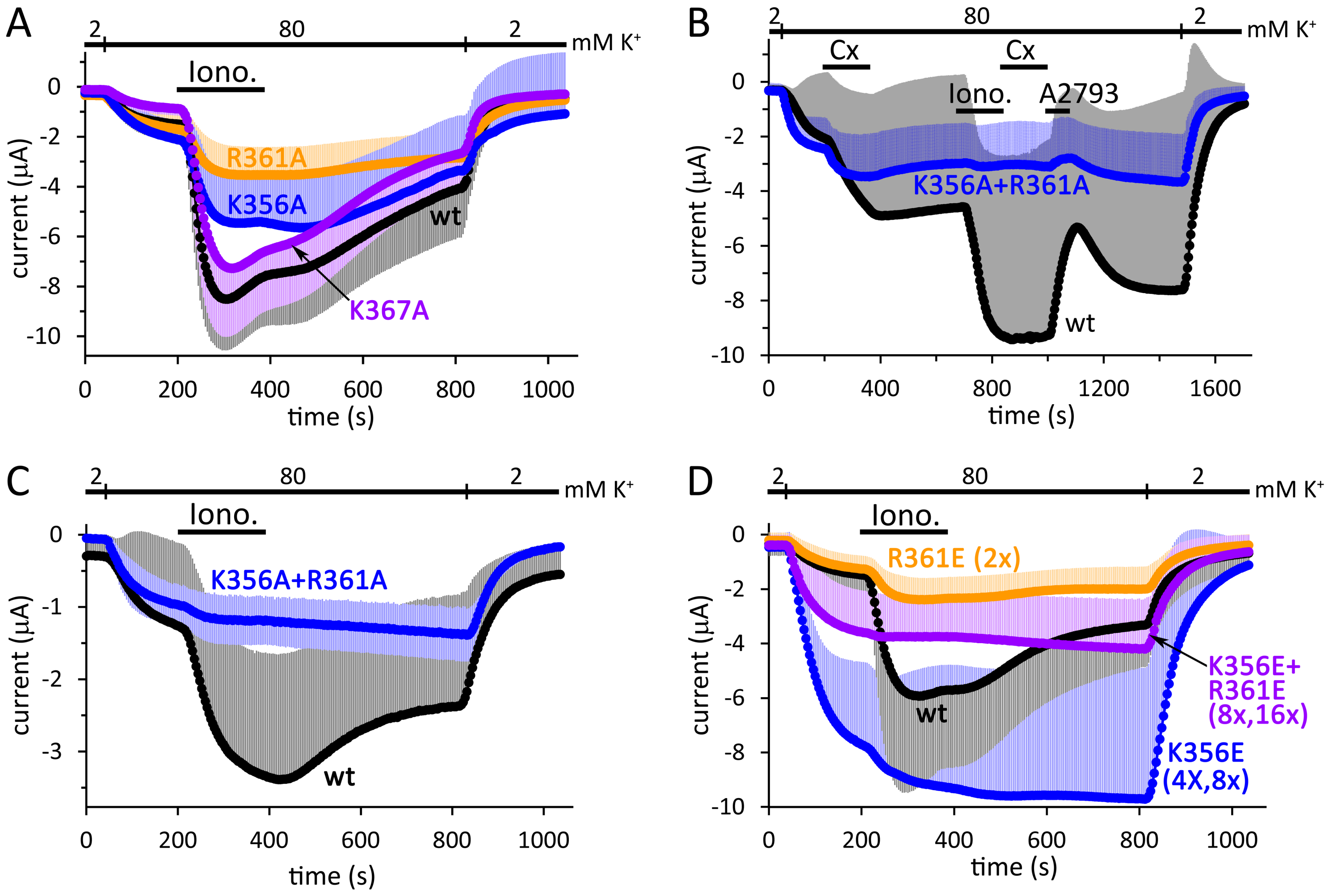

Figure S6. Average current data are shown from the conventional TEVC measurements of TRESK channels mutated at the K356, R361, or K367 residues, in accordance with Figure 5 of the main text.

Panels A and C correspond to the identically labelled panels of Figure 5 in the main text, but show average currents instead of the normalized curves. Panels B and D correspond to panels E and H of Figure 5 in the main text, respectively. (Sample numbers are given in the legend to Figure 5.) In panel D, twice as much (*2×*) cRNA of *R361E* mutant TRESK was microinjected as in the wild type (*wt*) group. Similarly, four or eight times more (*4×,8×*) cRNA of K356E mutant, and eight or sixteen times more (*8×,16×*) of the K356E+R361E double mutant channel was injected than in the control TRESK group.

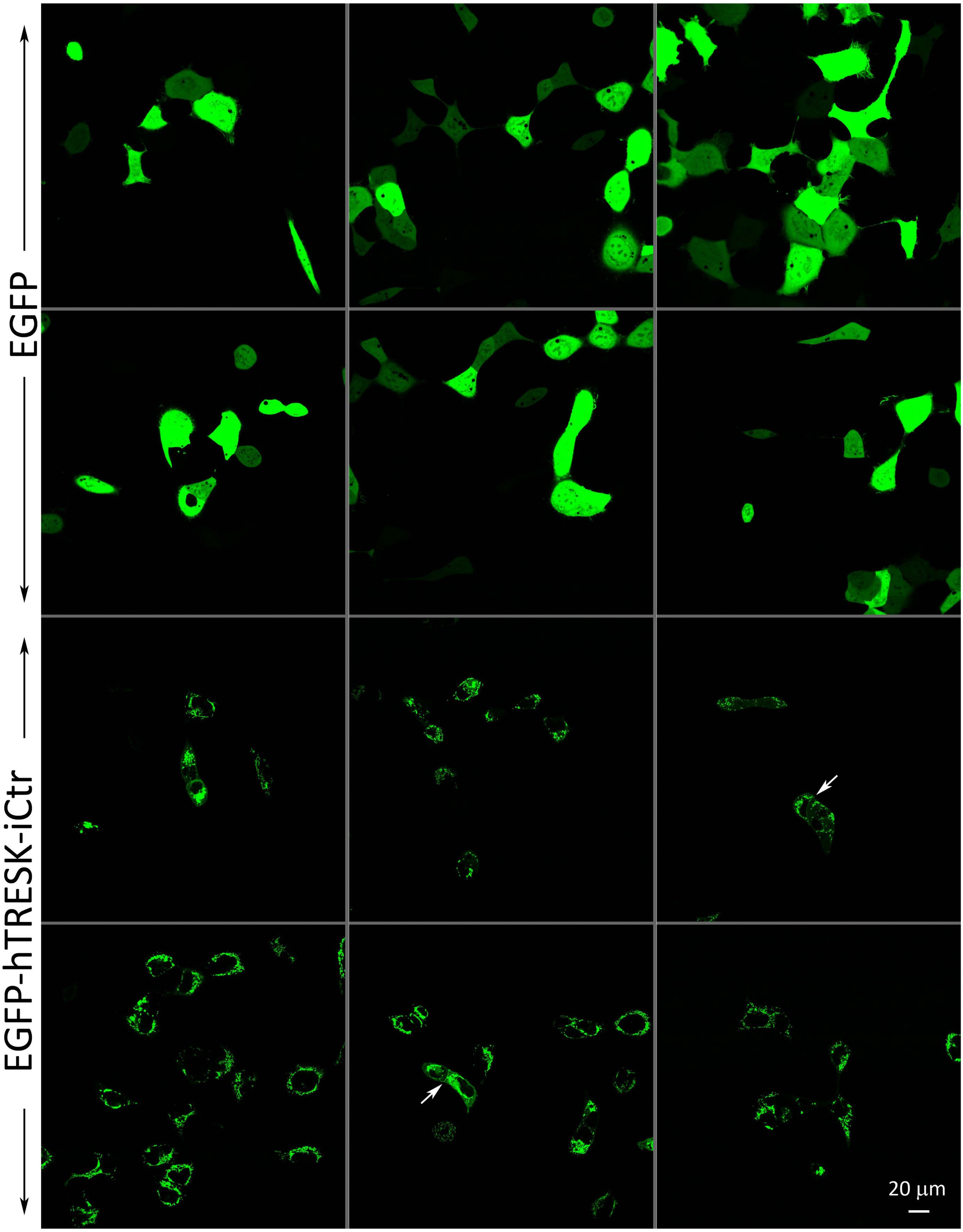

Figure S7. Confocal microscopy images showing the localization of EGFP fused to TRESK iCtr. In the upper *six images*, the cytoplasmic localization of enhanced green fluorescent protein (*EGFP*) is visible as a control, whereas the lower *six images* show the *EGFP-hTRESK-iCtr* construct, containing the intracellular C-terminal region (29 amino acids) of human TRESK fused to the C-terminus of EGFP. The fusion protein appears as a sharply localized and intense intracellular fluorescence with a possible additional accumulation in the plasma membrane of adjacent cells in rare instances (indicated with *white arrows*). The constructs were transiently expressed in HEK-293A cells, and all images were captured under identical conditions.

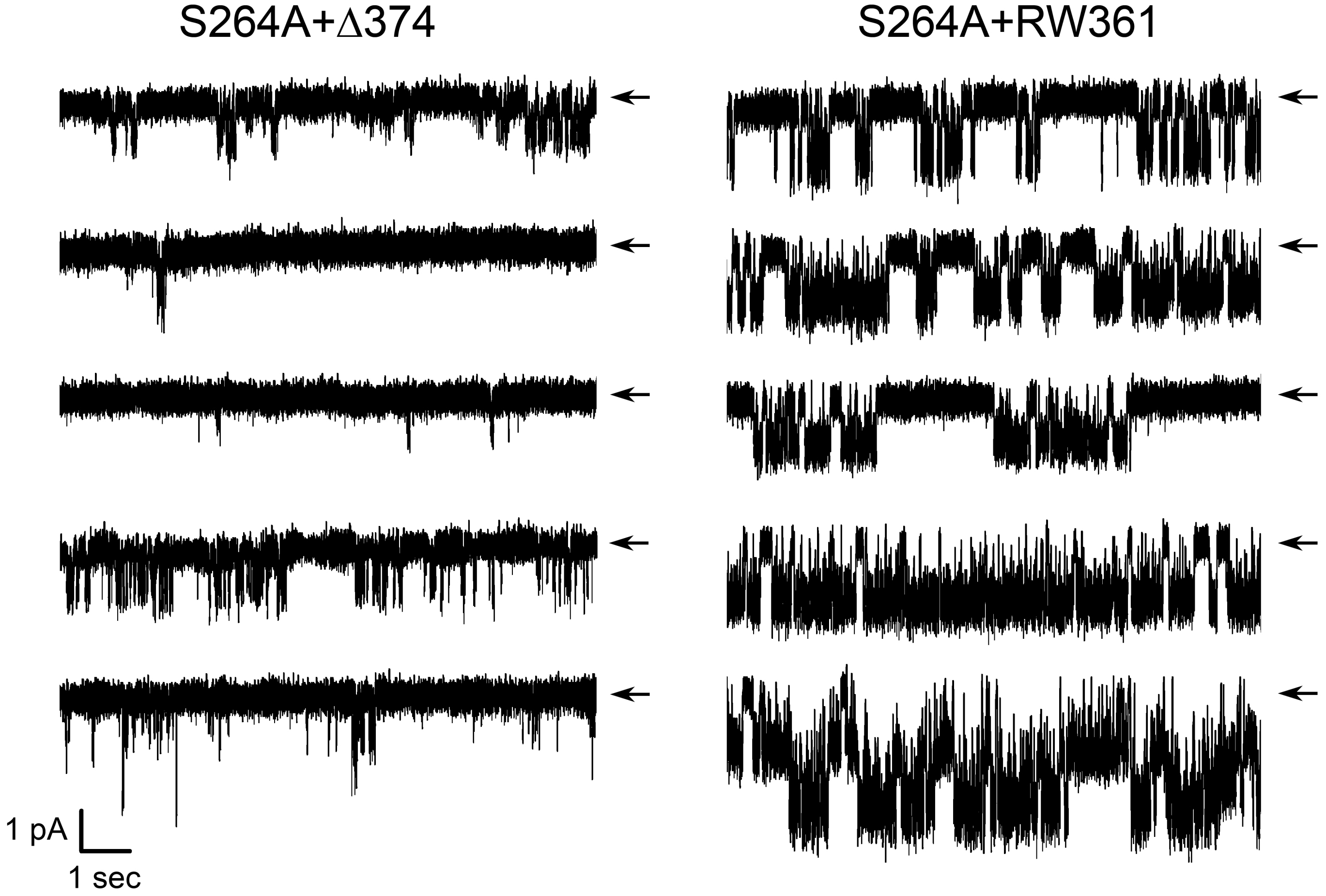

Figure S8. Representative single channel recordings of the S264A+Δ374 and S264A+RW361 versions of human TRESK.

Five recordings of the *S264A+Δ374* mutant TRESK channel are shown on the *left side*, whereas five recordings of *S264A+RW361* on the *right side* of the figure, as indicated *above the curves*. The membrane patch contained two channels in the *lower trace* of the *S264A+RW361* group, whereas the *upper four curves* display real single channel activity. At least two channels were present in the patch corresponding to the *lower trace* in the *S264A+Δ374* group, although the *upper recordings* might also contain multiple channels. All recordings show representative segments of single channel currents from different membrane patches. The method of measurement is described in Figure 12 of the main text, and the *bottom left* and *(from above) second right* recordings are shown in that figure as overall representatives. Zero current levels are indicated with *horizontal arrows*.

Figure S9.

Derivation of the ENaR equation

The macroscopic potassium current through the plasma membrane, I_K, is given by:

$$I_K = N i_K P_{oK} \tag{1}$$

where, N is the number of the ENaC-TRESK-ENaC complexes, i_K is the unitary conductance, and P_{oK} is the probability of the open state of the K^+ channel. (For simplicity, here we assumed that i_K and P_{oK} are the same for each K^+ channel, although this is not necessarily so under real whole cell conditions. There may be subconductance levels and different functional (e.g. phosphorylation) states. In this case, ENaR becomes proportional to the average of the $i_K P_{oK}$ products.)

Similarly, the macroscopic sodium current, I_{Na}, is calculated as:

$$I_{Na} = 2N i_{Na} P_{oNa} \tag{2}$$

where, 2N is the number of the ENaC pores in the N number of ENaC-TRESK-ENaC complexes, i_{Na} is the unitary conductance, and P_{oNa} is the probability of the open state of the Na^+ channel.

By applying the above formulas for I_K and I_{Na}, the ENaR value can be calculated according to the definition, and the number of channel complexes (N) cancels out from the equation:

$$ENaR = \frac{I_K}{I_{Na}} = \frac{N i_K P_{oK}}{2N i_{Na} P_{oNa}} = \frac{1}{2 i_{Na} P_{oNa}} i_K P_{oK} = \alpha i_K P_{oK} \tag{3}$$

where

$$\alpha = \frac{1}{2 i_{Na} P_{oNa}} \tag{4}$$

is constant.

Here we adopt the reasonable assumption that the unitary conductance and probability of the open state of the ENaC pores are independent from the mutations in the TRESK channel. Accordingly, α is equal in each group; it is a constant in the same cell preparation under identical experimental conditions. Note that the relationships of ENaR values are invariant to the exact stoichiometry of Na^+ and K^+ channel pores in the protein complex, as long as this stoichiometry is fixed in the different groups, because a different stoichiometry would only change the value "2" in the α constant (in eq. 4).

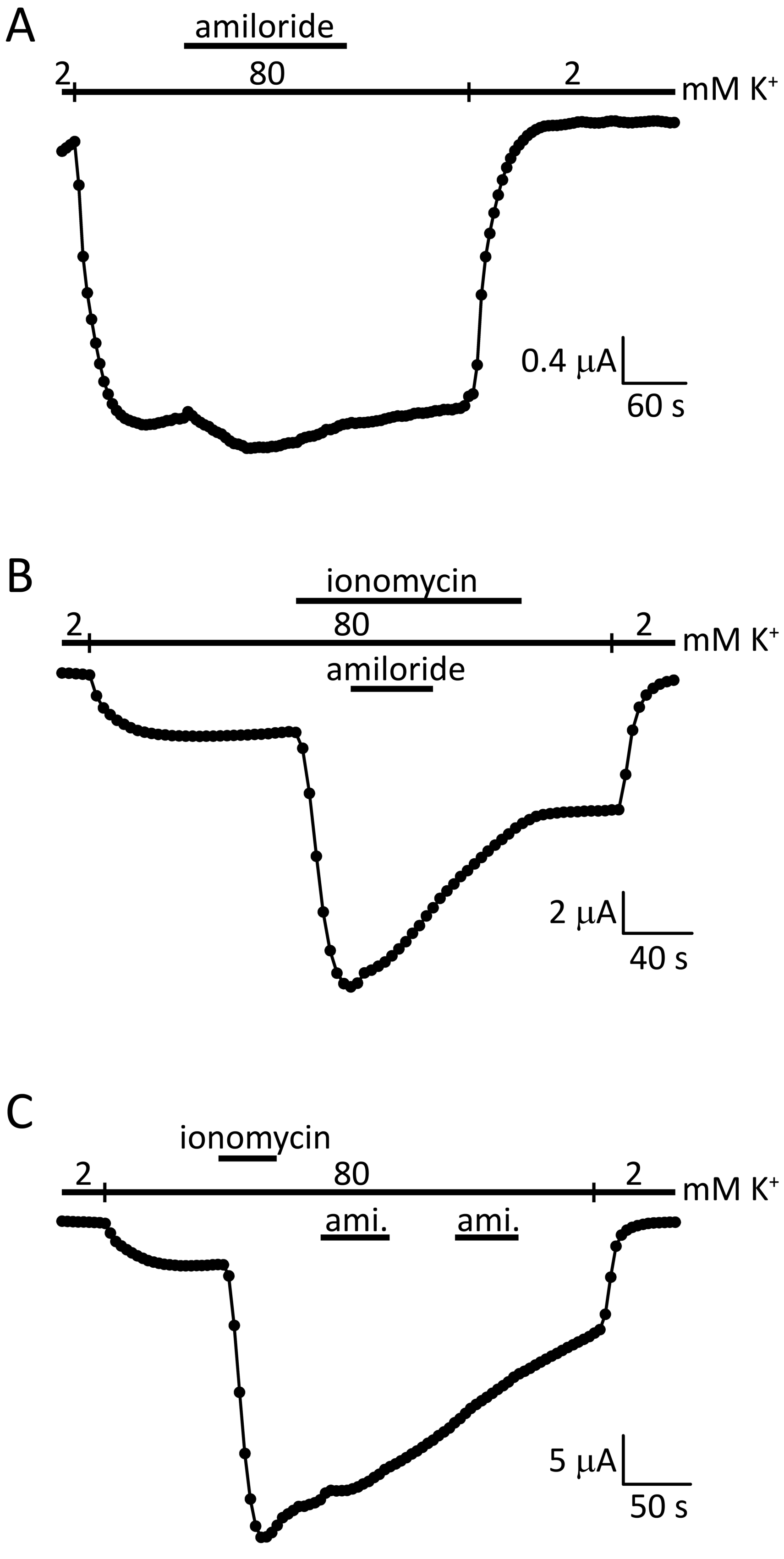

Figure S10. TRESK is not inhibited by amiloride.
A. Conventional TEVC measurement of the basal K^+ current in an oocyte expressing human TRESK. Amiloride (10 μM) was administered in the presence of high (80 mM) [K^+], as indicated *above the curve*. **B. and C.** Conventional TEVC recordings from two oocytes expressing TRESK, showing the lack of amiloride (*ami.*) effect on the stimulated channel, when this inhibitor was added after the application of *ionomycin,* as indicated *above the curves.* In *panel B*, the decrease of K^+ current in the maintained presence of ionomycin after the rapid activation of human TRESK, is a usual phenomenon.

Fig. S11. Results of the post hoc tests after ANOVA are summarized for all figures, as indicated above the tables. Tukey HSD post hoc test was used only after we found a statistically significant result in ANOVA (*not shown*). The factor codes (integers in the headers of the tables, *Var1*) correspond to the order of the groups in the graphs of the main text (or the *Supporting information* file). Statistica™ program outputs are given.

Fig. 2.C

Tukey HSD test; variable Var2 (Spreadsheet2)
Approximate Probabilities for Post Hoc Tests
Error: Between MS = .00226, df = 33.000

Var1	{1}	{2}	{3}
1		0.002665	0.911267
2	0.002665		0.001120
3	0.911267	0.001120	

Fig. 4.A (codes: 1:wt; 2:Δ374; 3:Δ366; 4:Δ357, K^+ currents at 360 sec)

Tukey HSD test; variable Var2 (Spreadsheet6)
Approximate Probabilities for Post Hoc Tests
Error: Between MS = 2997E4, df = 28.000

Var1	{1}	{2}	{3}	{4}
1		0.134468	0.002751	0.002641
2	0.134468		0.000174	0.000173
3	0.002751	0.000174		0.999999
4	0.002641	0.000173	0.999999	

Fig. 4.B

Tukey HSD test; variable Var2 (Spreadsheet1)
Approximate Probabilities for Post Hoc Tests
Error: Between MS = .00031, df = 55.000

Var1	{1}	{2}	{3}	{4}
1		0.000162	0.000162	0.001635
2	0.000162		0.999998	0.274264
3	0.000162	0.999998		0.265817
4	0.001635	0.274264	0.265817	

Fig. 5.B

Tukey HSD test; variable Var2 (Spreadsheet1)
Approximate Probabilities for Post Hoc Tests
Error: Between MS = 3.2155, df = 47.000

Var1	{1}	{2}	{3}	{4}
1		0.000170	0.000169	0.022309
2	0.000170		0.826561	0.000169
3	0.000169	0.826561		0.000169
4	0.022309	0.000169	0.000169	

Fig. S11. Continued (*second page*)

Fig. 5.I

Tukey HSD test; variable Var2 (Spreadsheet4)
Approximate Probabilities for Post Hoc Tests
Error: Between MS = 1.2105, df = 44.000

Var1	{1}	{2}	{3}	{4}
1		0.000169	0.000169	0.000169
2	0.000169		0.191853	0.990429
3	0.000169	0.191853		0.076483
4	0.000169	0.990429	0.076483	

Fig. 6.A

Tukey HSD test; variable Var2 (Spreadsheet1)
Approximate Probabilities for Post Hoc Tests
Error: Between MS = .00036, df = 65.000

Var1	{1}	{2}	{3}	{4}	{5}	{6}
1		0.948802	0.998537	0.000130	0.949843	0.981407
2	0.948802		0.787570	0.000130	1.000000	1.000000
3	0.998537	0.787570		0.000130	0.794353	0.902177
4	0.000130	0.000130	0.000130		0.000130	0.000130
5	0.949843	1.000000	0.794353	0.000130		1.000000
6	0.981407	1.000000	0.902177	0.000130	1.000000	

Fig. 6.C

Tukey HSD test; variable Var2 (Spreadsheet3)
Approximate Probabilities for Post Hoc Tests
Error: Between MS = .00064, df = 30.000

Var1	{1}	{2}	{3}	{4}
1		0.790349	0.645625	0.999977
2	0.790349		0.998555	0.804799
3	0.645625	0.998555		0.680780
4	0.999977	0.804799	0.680780	

Fig. 7.

Tukey HSD test; variable Var2 (Spreadsheet1)
Approximate Probabilities for Post Hoc Tests
Error: Between MS = .00142, df = 63.000

Var1	{1}	{2}	{3}	{4}	{5}
1		0.000131	0.999956	0.834286	0.132040
2	0.000131		0.000131	0.000145	0.003845
3	0.999956	0.000131		0.784899	0.114053
4	0.834286	0.000145	0.784899		0.681943
5	0.132040	0.003845	0.114053	0.681943	

Fig. S11. Continued (*third page*)

Fig. 8.

Tukey HSD test; variable Var2 (Spreadsheet2)
Approximate Probabilities for Post Hoc Tests
Error: Between MS = .00643, df = 61.000

Var1	{1}	{2}	{3}	{4}	{5}
1		0.999933	0.999911	0.948827	0.000133
2	0.999933		1.000000	0.893020	0.000133
3	0.999911	1.000000		0.914312	0.000133
4	0.948827	0.893020	0.914312		0.000133
5	0.000133	0.000133	0.000133	0.000133	

Fig. 9.A (codes: 1:wt; 2:356-6Kcaax; 3:367-6Kcaax; 4:375-6Kcaax, K^+ currents at 360 sec)

Tukey HSD test; variable Var2 (Spreadsheet13)
Approximate Probabilities for Post Hoc Tests
Error: Between MS = 4958E4, df = 32.000

Var1	{1}	{2}	{3}	{4}
1		0.089880	0.085317	0.000165
2	0.089880		0.999997	0.000165
3	0.085317	0.999997		0.000165
4	0.000165	0.000165	0.000165	

Fig. 9.B

Repeated Measures Analysis of Variance (Spreadsheet2)
Sigma-restricted parameterization
Effective hypothesis decomposition

	SS	Degr. of	MS	F	p
Intercept	0.457411	1	0.457411	208.9857	0.000000
"Var1"	0.251476	2	0.125738	57.4482	0.000000
Error	0.087549	40	0.002189		
IONO	0.081555	1	0.081555	51.1466	0.000000
IONO*"Var1"	0.097132	2	0.048566	30.4577	0.000000
Error	0.063782	40	0.001595		

Tukey HSD test; variable DV_1 (Spreadsheet2)
Approximate Probabilities for Post Hoc Tests
Error: Between; Within; Pooled MS = .00189, df = 78.074

	Var1	IONO	{1}	{2}	{3}	{4}	{5}	{6}
1	1	Var2		0.753106	0.999969	0.983445	0.067546	0.000125
2	1	Var3	0.753106		0.905389	0.994414	0.613020	0.000125
3	2	Var2	0.999969	0.905389		0.994741	0.117033	0.000125
4	2	Var3	0.983445	0.994414	0.994741		0.305746	0.000125
5	3	Var2	0.067546	0.613020	0.117033	0.305746		0.000139
6	3	Var3	0.000125	0.000125	0.000125	0.000125	0.000139	

Fig. S11. Continued (*fourth page*)

Fig. 11.A (codes: 1:wt; 2:RW361; 3:S264A; 4:S264A+RW361, K^+ currents at 360 sec)

Tukey HSD test; variable Var2 (Spreadsheet17)

Approximate Probabilities for Post Hoc Tests

Error: Between MS = 5458E3, df = 36.000

Var1	{1}	{2}	{3}	{4}
1		0.000282	0.896881	0.000339
2	0.000282		0.000593	0.995864
3	0.896881	0.000593		0.000779
4	0.000339	0.995864	0.000779	

Fig. 11.C (after logarithmic transformation)

Tukey HSD test; variable Var2 (Spreadsheet11)

Approximate Probabilities for Post Hoc Tests

Error: Between MS = .06591, df = 56.000

Var1	{1}	{2}	{3}	{4}
1		0.000158	0.000158	0.000158
2	0.000158		0.000158	0.000158
3	0.000158	0.000158		0.182164
4	0.000158	0.000158	0.182164	

Fig. S2.B

Multiple Comparisons p values (2-tailed); Var2 (Spreadsheet6)

Independent (grouping) variable: Var1

Kruskal-Wallis test: H (2, N= 21) =14.38281 p =.0008

Var1	1	2	3
1		0.001438	0.011753
2	0.001438		1.000000
3	0.011753	1.000000	

Fig. S5.

Tukey HSD test; variable Var2 (Spreadsheet1)

Approximate Probabilities for Post Hoc Tests

Error: Between MS = .00069, df = 41.000

Var1	{1}	{2}	{3}
1		0.000643	0.000120
2	0.000643		0.000986
3	0.000120	0.000986	
